# Theoretical Approach and Scale Construction of Patient Privacy Protection Behavior of Doctors in Public Medical Institutions in China: Pilot Development Study

**DOI:** 10.2196/39947

**Published:** 2022-12-14

**Authors:** Jie Xu, Lu Lu, Kaichen Xing, Huwei Shi, Ruiyao Chen, Yujun Yao, Sichen Liu, Zhongzhou Xiao, Xinwei Peng, Shuqing Luo, Yun Zhong

**Affiliations:** 1 Shanghai Artificial Intelligence Laboratory West Bank International Artificial Intelligence Center Shanghai China; 2 Université de Montpellier Montpellier France

**Keywords:** scale, Chinese public medical institutions, doctors’ protection behavior of patients’ privacy

## Abstract

**Background:**

Considering the high incidence of medical privacy disclosure, it is of vital importance to study doctors’ privacy protection behavior and its influencing factors.

**Objective:**

We aim to develop a scale for doctors’ protection of patients’ privacy in Chinese public medical institutions, following construction of a theoretical model framework through grounded theory, and subsequently to validate the scale to measure this protection behavior.

**Methods:**

Combined with the theoretical paradigm of protection motivation theory (PMT) and semistructured interview data, the grounded theory research method, followed by the Delphi expert and group discussion methods, a theoretical framework and initial scale for doctors in Chinese public medical institutions to protect patients' privacy was formed. The adjusted scale was collected online using a WeChat electronic survey measured using a 5-point Likert scale. Exploratory and confirmatory factor analysis (EFA and CFA) and tests to analyze reliability and validity were performed on the sample data. SPSS 19.0 and Amos 26.0 statistical analysis software were used for EFA and CFA of the sample data, respectively.

**Results:**

According to the internal logic of PMT, we developed a novel theoretical framework of a “storyline,” which was a process from being unaware of patients' privacy to having privacy protection behavior, that affected doctors' cognitive intermediary and changed the development of doctors' awareness, finally affecting actual privacy protection behavior in Chinese public medical institutions. Ultimately, we created a scale to measure 18 variables in the theoretical model, comprising 63 measurement items, with a total of 208 doctors participating in the scaling survey, who were predominantly educated to the master’s degree level (n=151, 72.6%). The department distribution was relatively balanced. Prior to EFA, the Kaiser-Meyer-Olkin (KMO) value was 0.702, indicating that the study was suitable for factor analysis. The minimum value of Cronbach *α* for each study variable was .754, which met the internal consistency requirements of the scale. The standard factor loading value of each potential measurement item in CFA had scores greater than 0.5, which signified that all the items in the scale could effectively converge to the corresponding potential variables.

**Conclusions:**

The theoretical framework and scale to assess doctors' patient protection behavior in public medical institutions in China ﬁlls a significant gap in the literature and can be used to further the current knowledge of physicians’ thought processes and adoption decisions.

## Introduction

### Background

Privacy disclosure [1] refers to the release, transfer, access, or disclosure of information in any way to an individual or group outside of the entity holding the information. According to a Verizon data disclosure survey, the medical industry is the only industry with an internal threat higher than an external threat, considering that a significant number of medical data leaks are associated with internal medical staff [2]. The high incidence of medical data [3-5] and patient information leakages may cause patient identity violations [6] and financial losses [7], alongside potentially more severe social effects. Currently, in China, organizations at all levels regulate the privacy protection behavior of medical staff by publishing relevant policies and setting privacy protection requirements for medical staff [8-10]. Generally, hospitals also have privacy disclosure restrictions medical practitioners must adhere to for patient privacy protection. However, even with multiple patient privacy protection requirements, the leakage of patient privacy remains frequent. Unfortunately, as those with direct contact with medical information, the negligence or improper behavior of doctors has become 1 of the primary reasons for this [11,12]. Therefore, it is vital to study doctors' privacy protection behavior in the hospital setting. Public medical institutions, which are government interventions in the medical market, are of universal significance worldwide. They provide inexpensive welfare services for the public rather than high-priced private medical services. Current studies focus on the primary influencing factors affecting doctors' motivation to comply with data protection [13], the influencing factors of electronic medical record (EMR) privacy protection by doctors [7], and the reaction mechanism of doctors toward patients' privacy protection requirements [14]. Furthermore, personal factors, including age, gender, educational background, professional title, working years, position, and understanding of laws and regulations [15,16], alongside environmental factors, such as a strict patient privacy protection system, systematic training, sufficient materials for patient privacy protection, and demonstrations by managers [17], all had an impact on doctors' privacy protection behavior toward patients. Currently, research into the privacy protection behavior of doctors, including the factors that influence behavior and intention, remains insufficient. Similarly, a theoretical basis and measurement scale of patient privacy protection behavior of doctors in public medical institutions in China are yet to be formed.

Consequently, this study aims to use the method of grounded theory to construct a theoretical model framework of the patient privacy protection behavior of doctors in public medical institutions in China. The measurement items of each variable were defined in combination with the results of coding analysis. The measurement scale was created, and the data were analyzed by exploratory factor analysis (EFA) and confirmatory factor analysis (CFA).

### Theory

Noar [18] proposed that the selection of a theoretical framework requires comparisons of multiple theoretical frameworks. The theory of planned behavior [13,19], protection motivation theory (PMT) [20], and the Health Belief Model [7] are often included in the theoretical basis of privacy protection. Comparison of these 3 theories shows that for the privacy protection behavior of doctors, fear of negativity is an important motivation for behavior change. Threat appraisal (TA), coping appraisal (CA), social norms (SN), and ethical personal characteristics are supposed to influence the development of doctors' awareness of the protection of patient privacy, thus leading to modifications in the actual behavior of doctors. The criteria for designating the theoretical framework were the related behavioral theories adopted by the current studies on privacy protection, followed by domestic and international studies on the influence factors of health care workers’ privacy protection behavior, in which the main influence factors should be contained in the selected dimensions. Based on the fear of negativity, the privacy protection behavior of doctors in public medical institutions in China alters the motivation of protection behavior through obtaining relevant cognitive information and thus affects the individual’s protection behavior. Consequently, the framework of PMT is in line with the theoretical paradigm of this study. PMT was developed using Health Belief Model by Rogers et al [21] in 1975. The factors affecting health-related behavior include perceived severity (PSE), perceived susceptibility (PSU), self-efficacy (SE), and response cost (RC), in addition to the object's perceived intrinsic rewards (IRE) and extrinsic rewards (ERE) [22,23]. However, patients’ privacy protection by doctors in public medical institutions in China is the otherness behavior of non-right holders. The effect of social norms, attitudes, personal characteristics, and other factors on behavioral intentions are not considered in PMT. The mechanism of privacy protection by public medical institution doctors in China is yet to be fully explored. Exploratory research was required to construct a theoretical model of this study.

## Methods

### Theoretical Construction

Considering the practical problem of how to promote the protection of patients' privacy in public medical institutions in China, we used the the theoretical paradigm of PMT. Through theoretical sampling, we selected representative interviewees and subsequently interviewed them using a semistructured interview. The interview period spanned from January 25 to February 25, 2022, which was a total of 32 days. The original data of grounded theory coding analysis was generated from the interviews. Subsequently, program-based grounded theory [24] was used to analyze the coding based on the original interview data and refine the scope of the study, alongside discussing the logical relationship between them and building the theoretical model framework [25-27].

### Scale Design and Optimization

**Figure 1:**
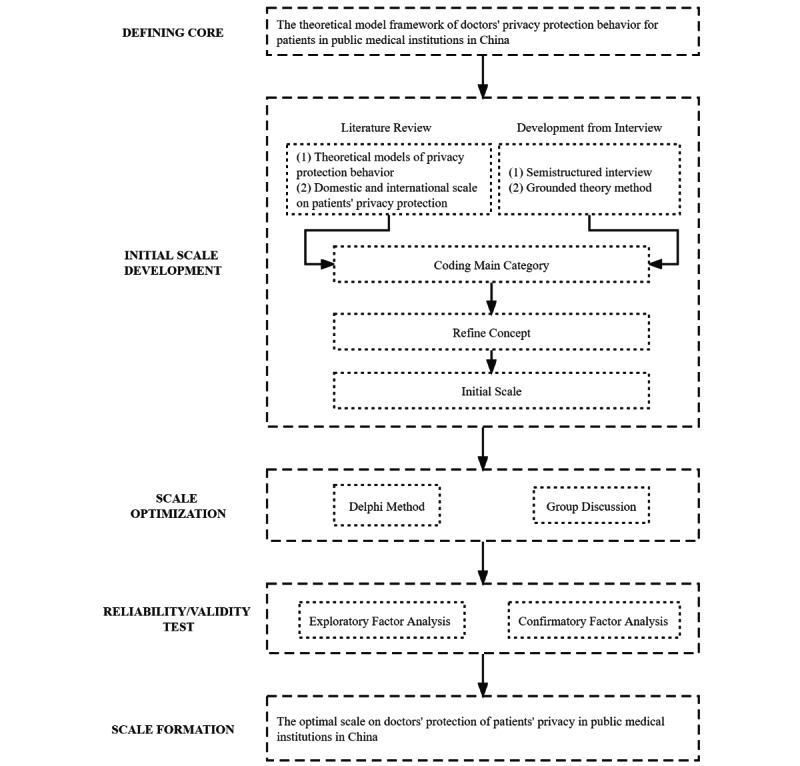
A Consolidated Standards of Reporting Trials (CONSORT) table of the process of the scale construction derived from Churchill's scale development.

Drawing on the methodology for scale development proposed by Churchill [28], a scale was designed based on the 3 principles of content, function, and overall uniformity. The process of scale construction is shown in Figure 1. Combined with the coding analysis results of the grounded theory method in the previous section, the key concept of interviews from the records of public medical institution doctors in China were extracted and the measurement items of the variables in this study were processed and separated. Regarding the initial scale, we had to make some adjustments to the items of the scale following the Delphi method [29] and a group discussion, including merging and deleting items with similar meanings in the scale, merging and adjusting items with an inclusion relationship, and adjusting the wording to ensure the semantic readability was concise and that the sentences were easy to understand to prevent misunderstandings.

### Sample Preparation

The criteria for recruitment of doctors to be interviewed were as follows: (1) serving public medical institutions in China, (2) definition of “doctors” corresponding to the indicators of the *China Health Statistical Yearbook*, and (3) voluntary participation in this study. The survey adopted convenience and snowball sampling and was sent in the form of an electronic survey scale through WeChat. Data collection ran from April 1 to 25, 2022. In this study, the explicative items of threat, coping, support, and ethical appraisal were measured in the form of a 5-point Likert scale [30]: 1=completely disagree, 2=disagree, 3=uncertain, 4=agree, and 5=fully agree. In addition, IRE, ERE, and RC were inversely assigned. The explained variables, such as consciousness formation, body privacy, information privacy, and related privacy, were measured and assigned “yes” or “no” using binary variables, corresponding to 1 or 0, respectively.

### Data Analysis

First, we conducted EFA, including a reliability evaluation using Cronbach *α*, which was the optimum method to evaluate the reliability of internal consistency. It is generally accepted that a Cronbach *α* score above .8 indicates excellent internal consistency, .6-.8 implies good consistency, and below .6 suggests poor internal consistency [31,32]. To ensure that the items involved in the measurement comprehensively and accurately measured the corresponding variables, we used EFA to assess the content validity of the scale. Although EFA is not an accurate method to test theoretical assumptions, it allowed us to draw conclusions regarding the construct validity of the proposed scale [33,34]. Prior to EFA, the Kaiser-Meyer-Olkin (KMO) and Bartlett sphericity test was conducted on the scale to determine whether the scale is suitable for factor analysis. According to the judgment standard of KMO values, when the KMO value is above 0.6, the scale can be subject to factor analysis, while if it is above 0.8, the scale is suitable for factor analysis [35]. The variance maximization rotation for principal component analysis of the measurement scale and the Kaiser normalization maximum variance method for rotation were used in the EFA of this study. The rotation was confirmed to have converged following 7 iterations. In this study, SPSS 19.0 statistical analysis software was used for EFA of the sample data.

CFA was helpful to verify whether the subordinate relationship between the items in the scale and the extracted factors were correct or whether there were any wrong attributions to dimension problems [36,37]. Concerning the CFA result, if the standardized factor load of the item is greater than 0.5, it is accepted that the item can converge to its corresponding latent variable. The maximum likelihood was used in the model estimation. The *χ*^2^ (*df*) value, the root-mean-square error of approximation (RMSEA), the standardized root-mean-square residual (SRMR), the Tucker-Lewis index (TLI), and the comparative ﬁt index (CFI) were calculated to evaluate the model ﬁt [38,39]. A model with good ﬁt is achieved if *χ*^2^ (*df*) is lower than 3 [40]. An RMSEA value below 0.05 indicates that the model is good [40]. SRMR values below 0.1 suggest that the model is acceptable [41]. The CFI and TLI values should be greater than 0.95 [42]. In addition, we evaluated the reliability of the scale by calculating the comprehensive reliability score [43,44] and analyzed both the convergence and discrimination effectiveness by comparing the average variance extracted (AVE) and the square correlation value [45,46]. If the variance of potential structure interpretation is greater than the variance according to measurement error (if the AVE value is higher than 0.5), the convergence effectiveness is clear [46]. If the AVE value is greater than the square correlation value, the discrimination effectiveness is obvious [46]. In conclusion, aggregate validity and discriminant validity are powerful indicators of structural validity [45,47]. Throughout this research, Amos 26.0 statistical analysis software was used for CFA of the samples.

### Ethical Considerations

Survey recipients were informed that participation was anonymous and voluntary, that all responses would be kept confidential, and that the collected data would be used for academic research only. The survey was approved by the Institutional Review Board at Huadong Sanatorium (approval no. (2022)13 of the Ethic Committee), and all participants provided written informed consent.

## Results

### Theoretical Model

**Figure 2:**
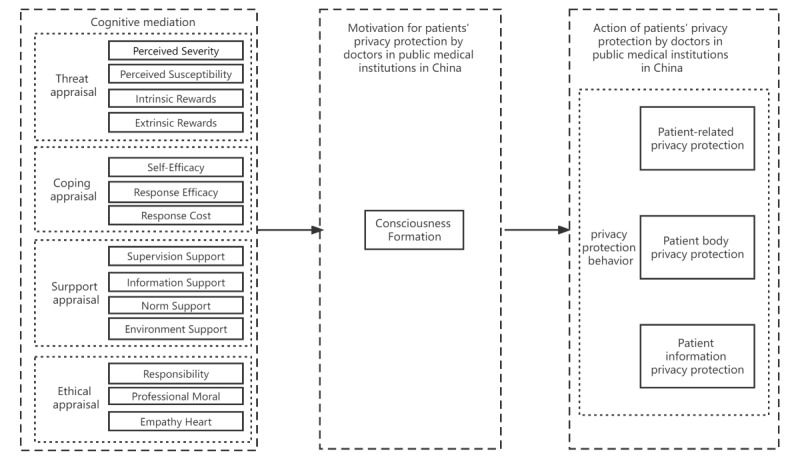
The theoretical model framework of doctors' privacy protection behavior for patients in public medical institutions in China.

In total, 26 public medical institution doctors in China were selected, 10 (38.5%) of whom had personal, in-depth interviews, while the remaining 16 (61.5%) had 2 online focus group interviews according to their time arrangement. Finally, we obtained 12 interview records of over 50,000 words. Based on coding analysis and the theoretical saturation test, the results are detailed in Multimedia Appendix 1. According to the internal logic of PMT, the cognitive intermediary of doctors in public medical institutions in China regarding patient privacy protection would affect the doctors' behavior by altering the development of their awareness of the protection of patient privacy. The protection of patients’ privacy under the awareness of public medical institutions would lead to modifications in the actual behavior of doctors. Consequently, in this study, we considered doctors in public medical institutions in China to move through a process, from being unaware of patients' privacy to having privacy protection behavior. We regarded that this process affected doctors' cognitive intermediary and changed the development of their awareness of patients' privacy protection in public medical institutions in China in order to affect actual privacy protection behavior. According to the logical relationship of this “storyline,” we developed a novel theoretical framework, which is the theoretical model framework of the mechanism of doctors' protection of patients' privacy in public medical institutions in China, as illustrated in Figure 2.

### Optimization of the Initial Scale

The results of the initial scale are displayed in Multimedia Appendix 2. A total of 15 items were corrected. Following the aforementioned correction and adjustment of measurement items, an initial scale to measure 18 direct measurement variables in the theoretical model of doctors' behavior mechanism of protecting patients' privacy in public medical institutions in China was formed. This also included 63 measurement items, which were coded. Tables 1-5 illustrates the specific codes and corresponding measurement items.

**Table 1:** Item setting of the initial scale for TA^a^.

Variable and code		Item
**PSE^b^**		
	PSE1	I think it is very serious and dangerous that the disclosure of patient privacy information will incur punishment by laws and regulations.
	PSE2	I think it is very serious and dangerous that the disclosure of patient privacy information will protect patients' rights and deepen the contradiction between doctors and patients.
	PSE3	I think it is very serious and dangerous that the disclosure of patient privacy information will incur punishment according to the hospital standard system.
**PSU^c^**		
	PSU1	I think that laws and regulations pay increasing attention to the protection of patients' privacy and have the tendency to make mandatory punishment measures for privacy disclosure.
	PSU2	I think that patients’ awareness of protecting rights is progressively becoming stronger, and the protection of personal privacy is paid increasing attention. The leakage of patient privacy will further deepen the contradiction between doctors and patients.
	PSU3	I think hospitals pay increasing attention to the privacy protection of patients, and the standards and systems will be more and more rigorous, and privacy disclosure incidents will be punishable.
**IRE^d^**		
	IRE1	I think that the disclosure of patients' privacy can be exchanged for certain financial returns.
	IRE2	I think it is inevitable that patient privacy will be leaked in the process of scientific research output.
	IRE3	I think meeting celebrities or attending new events at work will ‘get out’ on personal social platforms.
**ERE^e^**		
	ERE1	I've heard about the exchange of property through patient privacy information.
	ERE2	I hear that the easier it is for individuals or institutions to get patient data, the greater the output of scientific research.
	ERE3	I have heard that doctors have exposed some medical information or personal information about celebrities and related people on social platforms.

^a^TA: threat appraisal.

^b^PSE: perceived severity.

^c^PSU: perceived susceptibility.

^d^IRE: intrinsic rewards.

^e^ERE: extrinsic rewards.

**Table 2:** Item setting of the initial scale for CA^a^.

Variable and code			Item
**SE^b^**			
	SE1	I think it's easy for me to protect the privacy of patients.	
	SE2	I think it's convenient for me to protect the privacy of patients.	
	SE3	I have the ability to protect the privacy of patients from being disclosed.	
**RE^c^**			
	RE1	I think the doctors' protection measures to ensure the privacy of patients can effectively prevent the leakage of patients' privacy.	
	RE2	I think the privacy protection measures of doctors can keep patients' privacy in a safe environment.	
	RE3	I think the privacy protection measures of doctors for patients can better protect the privacy of patients.	
**RC^d^**			
	RC1	I think that paying attention to the protection of patients' privacy will affect the output of my overall scientific research results.	
	RC2	I think that paying attention to the privacy protection of patients will affect the development and efficiency of my clinical work and teaching.	
	RC3	I think that paying attention to the privacy protection of patients will increase my work pressure.	

^a^CA: coping appraisal.

^b^SE: self-efficacy.

^c^RE: response efficacy.

^d^RC: response cost.

**Table 3:** Item setting of the initial scale for SA^a^.

Variable and code			Item
**SS^b^**			
	SS1	I think the protection of patients' privacy needs the full-time supervision and management of a hospital department.	
	SS2	I think it is necessary for the hospital to regularly organize training and assessment according to the laws and regulations related to patient privacy protection.	
	SS3	I think it is necessary for the hospital to regularly organize training and assessment for the hospital system related to patient privacy protection and other contents related to patient privacy protection.	
**IS^c^**			
	IS1	I think it is necessary to use information technology, artificial intelligence, and other technologies to improve the information construction level of hospitals for patient privacy protection.	
	IS2	I think it is necessary to carry out reasonable authority management on the information system to protect the patient's private information.	
	IS3	I think it is necessary to impose reasonable data transmission restrictions on the information system to protect patients' private information.	
**NS^d^**			
	NS1	I think it is necessary to build a patient privacy protection system and carry it out effectively to ensure the rationalization process of patient privacy protection in doctors' work.	
	NS2	I think it is necessary to combine the patient privacy protection system with the doctor's daily work, so that the doctor's behavior of protecting the patient's privacy becomes a daily aspect of the work.	
	NS3	I think it is necessary to formulate a reasonable scientific research application system and conduct scientific research efficiently on the basis of legal and compliant patient privacy protection.	
**ES^e^**			
	ES1	I think improving the medical environment (such as independent consulting room, sound insulation treatment of consulting room) can better protect the privacy of patients.	
	ES2	I think it is necessary to maintain the order of medical treatment (for example, prevent irrelevant patients from gathering in the consulting room), which can better protect the privacy of patients.	
	ES3	I think facilities that provide patient privacy protection (such as curtains and privacy processing of bedside card information) can better protect patient privacy.	

^a^SA: support appraisal.

^b^SS: supervision support.

^c^IS: information support.

^d^NS: norm support.

^e^ES: environment support.

**Table 4:** Item setting of the initial scale for EA^a^.

Variable and code			Item
**RS^b^**			
	RS1	I think it is the duty of doctors to protect patients' privacy.	
	RS2	I believe that doctors should protect patients' privacy.	
	RS3	I think my sense of responsibility urges me to protect patients' privacy in my daily work.	
**PM^c^**			
	PM1	I think doctors' protection of patients' privacy is a requirement of their own professional ethics.	
	PM2	I think my sense of professional ethics urges me to protect patients' privacy in my daily work.	
	PM3	From education to work, the protection of patients' private information is a professional ethic repeatedly emphasized by doctors.	
**EH^d^**			
	EH1	I think doctors should consider the harm of privacy information disclosure from the perspective of patients, to become more aware of protecting the privacy of patients.	
	EH2	I have had a personal information disclosure experience as a patient, so I am more aware of protecting the privacy of patients.	
	EH3	I think that I can ‘push myself to others’ to protect my patients' privacy in my daily work.	

^a^EA: ethical appraisal.

^b^RS: responsibility.

^c^PM: professional moral.

^d^EH: empathy heart.

**Table 5:** Item setting of the initial scale for CF^a^, BP^b^, IP^c^, and RP^d^.

Variable and code			Item
**CF**			
	CF1	I think I have developed a sense of privacy protection in my clinical work.	
	CF2	I think I have formed a sense of privacy protection in my teaching.	
	CF3	I think I have formed a sense of privacy protection in my own research work.	
**BP**			
	BP1	Protect the patient's privacy during surgery or examination, such as curtain pulling and preventing a third party from breaking in.	
	BP2	Effectively block the privacy of patients during live operations.	
	BP3	Medical observation or teaching requires the consent of the patient.	
	BP4	No illegal touch or peek at the patient's privacy.	
**IP**			
	IP1	In the situations of outpatient, ward check, case discussion, medical education and observation, the patient shall obtain the consent of the patient himself and take confidentiality measures. The privacy information of the patient shall not be publicized or publicly discussed orally, including the personal information and disease information with identifiable characteristics, such as avoiding calling the full name of the patient loudly, avoiding ‘listening’ or ‘breaking in’ by people other than patients without the consent of the patient.	
	IP2	In the face of the condition inquiry, strictly confirm and ask the status of the patient’s condition personnel, confirm as me or with my consent.	
	IP3	For patients with special conditions (for example infectious diseases involving privacy), it is necessary to talk to the patients individually.	
	IP4	Deliberately disclose and disseminate the privacy of patients without using their duties, such as taking the bedside card test sheets of celebrities to the internet.	
	IP5	Protect medical documents such as inspection and medical records without random placing, damage, loss, and prevent theft and being wrongly picked up.	
	IP6	Under the unnecessary diagnosis and treatment process, without the consent of the patient, the medical documents shall not be checked, copied, or borrowed during the hospitalization of the patient.	
	IP7	Use personal information system account number as required, and login to view patient information without borrowing non-authorised people.	
	IP8	Not disclose the privacy information of the patient for any benefit reasons to obtain business, advertise or defraud.	
	IP9	When leaving the office seat, protect the pages with patient privacy information and lock the screen of the computer.	
	IP10	Scientific research, including the mining of electronic medical record information, whether it is the steps of data acquisition, viewing, processing or analysis, is strictly done to de privacy.	
	IP11	In the form of talks or written (case discussion, writing medical treatises, scientific research papers), for example, when communicating and learning on medical social network platform to share typical cases, do well in privacy treatment.	
**RP**			
	RP1	Do not disclose information about family members and other personal relationships of any patient.	
	RP2	Do not disclose family members and other personal relationship information of any patient on social platforms.	
	RP3	Do not verbally promote or publicly discuss family members and other personal relationship information of any patient.	

^a^CF: consciousness formation.

^b^BP: body privacy.

^c^IP: information privacy.

^d^RP: related privacy.

### Descriptive Analysis Results

The survey we issued was scanned a total of 278 times, and 208 valid questionnaires were identified following recovery; thus, the effective recovery rate was 74.8%. The gender ratio of men to women was relatively balanced, with 46.6% (n=97) of the participants being men. The majority of the respondents were 26-35 years old, accounting for 63.9% (n=133) of the total sample. The respondents were predominantly educated to the master’s degree level, accounting for 72.6% (n=151), which was consistent with the general education background of doctors. The department distribution was relatively balanced. Regarding urban distribution, Shanghai accounted for the highest proportion (n=131, 63.0%) due to convenience sampling. The detailed characteristics of the pretest samples are displayed in Table 6.

**Table 6:** Statistical results of sample characteristics.

Sample characteristics and measurement items			Sample size (N=208), n (%)
**Gender**			
	Male	97 (46.6)	
	Female	111 (53.4)	
**Age (years)**			
	18-25	9 (4.3)	
	26-35	133 (63.9)	
	36-50	61 (29.3)	
	>50	5 (2.4)	
**Educational background**			
	Undergraduate	41 (19.7)	
	Master	151 (72.6)	
	Doctor	16 (7.7)	
**Department**			
	Otorhinolaryngology	5 (2.4)	
	Infectious diseases	5 (2.4)	
	Pulmonology	16 (7.7)	
	Severe medicine	9 (4.3)	
	Clinical laboratory	5 (2.4)	
	Endocrinology	5 (2.4)	
	Anesthesiology	24 (11.5)	
	Pediatrics	23 (11.1)	
	Internal medicine	23 (11.1)	
	Burns and plastic surgery	5 (2.4)	
	Internal medicine: cardiovascular	16 (7.7)	
	Surgery	9 (4.3)	
	Ophthalmology	9 (4.3)	
	Medical service	9 (4.3)	
	Imaging	18 (8.7)	
	Oncology	9 (4.3)	
	Dental	18 (8.7)	
**City**			
	Beijing	20 (9.6)	
	Changzhou, Jiangsu Province	5 (2.4)	
	Huai'an, Jiangsu Province	5 (2.4)	
	Nanjing, Jiangsu Province	5 (2.4)	
	Nantong, Jiangsu Province	5 (2.4)	
	Wuxi, Jiangsu Province	32 (15.4)	
	Zhenjiang, Jiangsu Province	5 (2.4)	
	Shanghai	131 (63.0)	

### Exploratory and Verifiable Analysis

The Cronbach *α* of the whole scale was determined to be .768 by calculating the consistency coefficient of the scale, which was between 0.6 and 0.8, indicating that the scale possessed good internal consistency. According to the results illustrated in Table 7, the KMO value was greater than 0.6, indicating that the study is suitable for factor analysis, theoretically.

Following the factor analysis operation, 18 common factors were screened. The cumulative interpretation total variance of factor analysis was 71.49%, implying that this research had good explanatory ability. The contribution of single-factor variance was less than 40%, demonstrating that this scale could exclude any possible homologous deviation [48]. In general, this scale conformed to the preassumed theoretical structure and possessed good content validity. From the factor loading of each dimension, it was clear that the measurement items were independent of common factors, and the load was above 0.7, far greater than the standard of 0.4. Conversely, the absolute value of the load of the 18-factor measurement items on other factors was below 0.4. This indicates that the items of variables in this study could both effectively converge on their own common factors and effectively be different from other common factors. The minimum value of the Cronbach *α* for each study variable was .754; thus, the coefficients for each study variable were between 0.6 and 0.8, which met the internal consistency requirements of the scale. Consequently, the scale in this study could be considered to have good convergence validity and internal consistency [49]. The results are stated in Multimedia Appendix 3.

According to the fitting results of the model data in Table 8, most of the fitting indexes met the requirements of a critical value, indicating that the confirmatory factor model was well fit. According to the results of CFA in Table 9, the standard factor loading value of each potential measurement item in this study had scores greater than 0.5. The critical ratio (CR) values were greater than 7, and all had statistical significance levels within the range of *P*<.001. All the composite reliability (CPR) values were greater than 0.7, and the value of AVE scores were in excess of 0.5. These indexes were in accordance with the standards, which signified that all the items in the scale can effectively converge to the corresponding potential variables. The data displayed in Table 10 show that the value of the square root of the AVE of each variable was greater than 0.7. The score was significantly greater than the correlation value of the row and column in which it was located, namely the value below the diagonal. This demonstrated that there were significant differences among variables and confirmed that the scale in this study has good discriminative validity.

**Table 7:** Results of KMO^a^ and Bartlett sphericity test.

Variable			Value
KMO metric for sufficient sampling			0.702
**Bartlett sphericity test**			
	Approximate *χ*^2^ (*df*)	6130.640 (1953)	
	Significance	0.000	

^a^KMO: Kaiser-Meyer-Olkin.

**Table 8:** Judgment of fitting indexes of the CFA^a^ model.

Model fitting index	Critical value	Model fitting index value	Model fit judgment
*χ*^2^/*df*	<3	1.138	Yes
RMSEA^b^	<0.08	0.026	Yes
SRMR^c^	<0.1	0.0474	Yes
CFI^d^	>0.95	0.951	Yes
TLI^e^	＞0.95	0.945	Yes

^a^CFA: confirmatory factor analysis.

^b^RMSEA: root-mean-square error of approximation.

^c^SRMR: standardized root-mean-square residual.

^d^CFI: comparative fit index.

^e^TLI: Tucker-Lewis index.

**Table 9:** Results of validation factor analysis.

Variable and item		Standard factor load	SE	CR^a^	*P* value
**PSE^b^ (AVE^c^=0.301, CPR^d^=0.819)**					
	PSE1	0.760	N/A^e^	N/A	N/A
	PSE2	0.794	0.095	9.997	<.001
	PSE3	0.771	0.095	9.869	<.001
**PSU^f^ (AVE=0.580, CPR=0.805)**					
	PSU1	0.720	N/A	N/A	N/A
	PSU2	0.752	0.112	9.199	<.001
	PSU3	0.810	0.119	9.454	<.001
**IRE^g^ (AVE=0.567, CPR=0.797)**					
	IRE1	0.778	N/A	N/A	N/A
	IRE2	0.708	0.101	8.951	<.001
	IRE3	0.770	0.130	9.341	<.001
**ERE^h^ (AVE=0.541, CPR=0.780)**					
	ERE1	0.706	N/A	N/A	N/A
	ERE2	0.747	0.149	8.513	<.001
	ERE3	0.753	0.133	8.535	<.001
**SE^i^ (AVE=0.603, CPR=0.819)**					
	SE1	0.697	N/A	N/A	N/A
	SE2	0.841	0.122	9.699	<.001
	SE3	0.784	0.111	9.515	<.001
**RE^j^ (AVE=0.569, CPR=0.798)**					
	RE1	0.799	N/A	N/A	N/A
	RE2	0.792	0.110	9.441	<.001
	RE3	0.665	0.100	8.641	<.001
**RC^k^ (AVE=0.567, CPR=0.796)**					
	RC1	0.702	N/A	N/A	N/A
	RC2	0.731	0.137	8.753	<.001
	RC3	0.820	0.145	9.048	<.001
**SS^l^ (AVE=0.532, CPR=0.773)**					
	SS1	0.719	N/A	N/A	N/A
	SS2	0.769	0.132	8.670	<.001
	SS3	0.699	0.125	8.290	<.001
**IS^m^ (AVE=0.568, CPR=0.797)**					
	IS1	0.719	N/A	N/A	N/A
	IS2	0.783	0.118	9.266	<.001
	IS3	0.757	0.108	9.124	<.001
**NS^n^ (AVE=0.586, CPR=0.809)**					
	NS1	0.770	N/A	N/A	N/A
	NS2	0.768	0.104	9.694	<.001
	NS3	0.758	0.100	9.630	<.001
**ES^o^ (AVE=0.567, CPR=0.797)**					
	ES1	0.768	N/A	N/A	N/A
	ES2	0.737	0.098	9.329	<.001
	ES3	0.753	0.095	9.448	<.001
**RS^p^ (AVE=0.547, CPR=0.784)**					
	RS1	0.719	N/A	N/A	N/A
	RS2	0.770	0.108	8.685	<.001
	RS3	0.729	0.120	8.524	<.001
**PM^q^ (AVE=0.545, CPR=0.782)**					
	PM1	0.695	N/A	N/A	N/A
	PM2	0.776	0.131	8.427	<.001
	PM3	0.742	0.122	8.350	<.001
**EH^r^ (AVE=0.539, CPR=0.778)**					
	EH1	0.722	N/A	N/A	N/A
	EH2	0.748	0.094	8.471	<.001
	EH3	0.732	0.097	8.407	<.001
**CF^s^ (AVE=0.513, CPR=0.757)**					
	CF1	0.801	N/A	N/A	N/A
	CF2	0.610	0.10	7.074	<.001
	CF3	0.724	0.122	7.480	<.001
**BP^t^ (AVE=0.552, CPR=0.831)**					
	BP1	0.701	N/A	N/A	N/A
	BP2	0.736	0.111	9.072	<.001
	BP3	0.792	0.119	9.539	<.001
	BP4	0.740	0.116	9.110	<.001
**IP^u^ (AVE=0.532, CPR=0.926)**					
	IP1	0.743	N/A	N/A	N/A
	IP2	0.718	0.094	10.391	<.001
	IP3	0.717	0.094	10.381	<.001
	IP4	0.723	0.094	10.468	<.001
	IP5	0.744	0.090	10.796	<.001
	IP6	0.806	0.095	11.802	<.001
	IP7	0.733	0.094	10.632	<.001
	IP8	0.67	0.091	9.703	<.001
	IP9	0.765	0.091	11.136	<.001
	IP10	0.704	0.093	10.171	<.001
	IP11	0.689	0.089	9.942	<.001
**RP^v^ (AVE=0.574, CPR=0.802)**					
	RP1	0.736	N/A	N/A	N/A
	RP2	0.781	0.116	9.043	<.001
	RP3	0.756	0.110	8.984	<.001

^a^CR: critical ratio.

^b^PSE: perceived severity.

^c^AVE: average variance extracted.

^d^CPR: composite reliability.

^e^N/A: not applicable.

^f^PSU: perceived susceptibility.

^g^IRE: intrinsic rewards.

^h^ERE: extrinsic rewards.

^i^SE: self-efficacy.

^j^RE: response efficacy.

^k^RC: response cost.

^l^SS: supervision support.

^m^IS: information support.

^n^NS: norm support.

^o^ES: environment support.

^p^RS: responsibility.

^q^PM: professional moral.

^r^EH: empathy heart.

^s^CF: consciousness formation.

^t^BP: body privacy.

^u^IP: information privacy.

^v^RP: related privacy.

**Table 10:** Results of validity test.

Variables	RP^a^	IP^b^	BP^c^	CF^d^	EH^e^	PM^f^	RS^g^	ES^h^	NS^i^	IS^j^	SS^k^	RC^l^	RE^m^	SE^n^	ERE^o^	IRE^p^	PSU^q^	PSE^r^
RP	*0.758^s^*	N/A^t^	N/A	N/A	N/A	N/A	N/A	N/A	N/A	N/A	N/A	N/A	N/A	N/A	N/A	N/A	N/A	N/A
IP	0.046	*0.730^s^*	N/A	N/A	N/A	N/A	N/A	N/A	N/A	N/A	N/A	N/A	N/A	N/A	N/A	N/A	N/A	N/A
BP	–0.145	0.015	*0.743^s^*	N/A	N/A	N/A	N/A	N/A	N/A	N/A	N/A	N/A	N/A	N/A	N/A	N/A	N/A	N/A
CF	–0.110	–0.069	0.045	*0.716^s^*	N/A	N/A	N/A	N/A	N/A	N/A	N/A	N/A	N/A	N/A	N/A	N/A	N/A	N/A
EH	0.010	–0.006	0.105	–0.001	*0.734^s^*	N/A	N/A	N/A	N/A	N/A	N/A	N/A	N/A	N/A	N/A	N/A	N/A	N/A
PM	–0.021	0.079	–0.029	–0.041	0.336	*0.738^s^*	N/A	N/A	N/A	N/A	N/A	N/A	N/A	N/A	N/A	N/A	N/A	N/A
RS	0.058	–0.038	0.034	–0.034	0.436	0.357	*0.740^s^*	N/A	N/A	N/A	N/A	N/A	N/A	N/A	N/A	N/A	N/A	N/A
ES	0.066	0.044	–0.026	–0.027	–0.062	–0.032	–0.056	*0.753^s^*	N/A	N/A	N/A	N/A	N/A	N/A	N/A	N/A	N/A	N/A
NS	–0.036	–0.010	–0.047	–0.053	–0.107	0.028	–0.116	0.425	*0.765^s^*	N/A	N/A	N/A	N/A	N/A	N/A	N/A	N/A	N/A
IS	–0.104	–0.012	0.047	–0.154	0.055	0.002	–0.013	0.489	0.408	*0.753^s^*	N/A	N/A	N/A	N/A	N/A	N/A	N/A	N/A
SS	0.008	–0.019	–0.009	–0.014	–0.075	0.038	–0.160	0.482	0.396	0.451	*0.730^s^*	N/A	N/A	N/A	N/A	N/A	N/A	N/A
RC	–0.073	–0.010	0.062	0.025	–0.021	0.035	0.060	–0.054	0.060	–0.013	0.008	*0.753^s^*	N/A	N/A	N/A	N/A	N/A	N/A
RE	–0.056	0.053	0.035	0.011	–0.041	0.027	0.009	–0.017	0.108	0.058	0.104	0.355	*0.755^s^*	N/A	N/A	N/A	N/A	N/A
SE	–0.046	0.005	–0.012	–0.043	0.013	0.078	–0.014	–0.051	0.001	0.038	0.112	0.458	0.350	*0.776^s^*	N/A	N/A	N/A	N/A
ERE	0.043	0.012	0.106	–0.068	0.039	0.053	0.076	0.014	–0.020	0.028	–0.001	–0.022	0.001	–0.080	*0.736^s^*	N/A	N/A	N/A
IRE	0.049	0.068	0.103	0.071	–0.017	0.083	–0.021	0.060	0.035	0.007	0.076	–0.029	0.010	–0.124	0.392	*0.753^s^*	N/A	N/A
PSU	0.118	0.017	0.066	–0.002	–0.081	0.056	0.002	–0.003	–0.028	0.023	–0.065	0.030	0.079	–0.041	0.438	0.369	*0.762^s^*	N/A
PSE	0.108	0.050	0.060	–0.032	–0.071	0.047	0.008	–0.008	0.001	–0.014	0.028	0.030	0.020	0.036	0.395	0.335	0.372	*0.775^s^*

^a^RP: related privacy.

^b^IP: information privacy.

^c^BP: body privacy.

^d^CF: consciousness formation.

^e^EH: empathy heart.

^f^PM: professional moral.

^g^RS: responsibility.

^h^ES: environment support.

^i^NS: norm support.

^j^IS: information support.

^k^SS: supervision support.

^l^RC: response cost.

^m^RE: response efficacy.

^n^SE: self-efficacy.

^o^ERE: extrinsic rewards.

^p^IRE: intrinsic rewards.

^q^PSU: perceived susceptibility.

^r^PSE: perceived severity.

^s^Italicized values are significant.

^t^N/A: not applicable.

### Scale Determination

We ultimately designed a scale with high reliability and validity and a correct subordination structure between factors. The scale determined 63 measurement items around 18 direct measurement variables in the theoretical model of this study to create a formal scale for this study. The specific contents are illustrated in Table 11.

**Table 11:** Measurement items of the formal scale of this study.

Variable and code		Measurement item
**PSE^a^**		
	PSE1	I think it is very serious and dangerous that the disclosure of patient privacy information will incur punishment by laws and regulations.
	PSE2	I think it is very serious and dangerous that the disclosure of patient privacy information will protect patients' rights and deepen the contradiction between doctors and patients.
	PSE3	I think it is very serious and dangerous that the disclosure of patient privacy information will incur punishment according to the hospital standard system.
**PSU^b^**		
	PSU1	I think that laws and regulations pay increasing attention to the protection of patients' privacy and have the tendency to make mandatory punishment measures for privacy disclosure.
	PSU2	I think that patients’ awareness of protecting rights is progressively becoming stronger, and the protection of personal privacy is paid increasing attention. The leakage of patient privacy will further deepen the contradiction between doctors and patients.
	PSU3	I think hospitals pay increasing attention to the privacy protection of patients, and the standards and systems will be more and more rigorous, and privacy disclosure incidents will be punishable.
**IRE^c^**		
	IRE1	I think that the disclosure of patients' privacy can be exchanged for certain financial returns.
	IRE2	I think it is inevitable that patient privacy will be leaked in the process of scientific research output.
	IRE3	I think meeting celebrities or attending new events at work will ‘get out’ on personal social platforms.
**ERE^d^**		
	ERE1	I've heard about the exchange of property through patient privacy information.
	ERE2	I hear that the easier it is for individuals or institutions to get patient data, the greater the output of scientific research.
	ERE3	I have heard that doctors have exposed some medical information or personal information about celebrities and related people on social platforms.
**SE^e^**		
	SE1	I think it's easy for me to protect the privacy of patients.
	SE2	I think it's convenient for me to protect the privacy of patients.
	SE3	I have the ability to protect the privacy of patients from being disclosed.
**RE^f^**		
	RE1	I think the doctors' protection measures to ensure the privacy of patients can effectively prevent the leakage of patients' privacy.
	RE2	I think the privacy protection measures of doctors can keep patients' privacy in a safe environment.
	RE3	I think the privacy protection measures of doctors for patients can better protect the privacy of patients.
**RC^g^**		
	RC1	I think that paying attention to the protection of patients' privacy will affect the output of my overall scientific research results.
	RC2	I think that paying attention to the privacy protection of patients will affect the development and efficiency of my clinical work and teaching.
	RC3	I think that paying attention to the privacy protection of patients will increase my work pressure.
**SS^h^**		
	SS1	I think the protection of patients' privacy needs the full-time supervision and management of a hospital department.
	SS2	I think it is necessary for the hospital to regularly organize training and assessment according to the laws and regulations related to patient privacy protection.
	SS3	I think it is necessary for the hospital to regularly organize training and assessment for the hospital system related to patient privacy protection and other contents related to patient privacy protection.
**IS^i^**		
	IS1	I think it is necessary to use information technology, artificial intelligence, and other technologies to improve the information construction level of hospitals for patient privacy protection.
	IS2	I think it is necessary to carry out reasonable authority management on the information system to protect the patient's private information.
	IS3	I think it is necessary to impose reasonable data transmission restrictions on the information system to protect patients’ private information.
**NS^j^**		
	NS1	I think it is necessary to build a patient privacy protection system and carry it out effectively to ensure the rationalization process of patient privacy protection in doctors' work.
	NS2	I think it is necessary to combine the patient privacy protection system with the doctor's daily work, so that the doctor's behavior of protecting the patient's privacy becomes a daily aspect of the work.
	NS3	I think it is necessary to formulate a reasonable scientific research application system and conduct scientific research efficiently on the basis of legal and compliant patient privacy protection.
**ES^k^**		
	ES1	I think improving the medical environment (such as independent consulting room, sound insulation treatment of consulting room) can better protect the privacy of patients.
	ES2	I think it is necessary to maintain the order of medical treatment (for example, prevent irrelevant patients from gathering in the consulting room), which can better protect the privacy of patients.
	ES3	I think facilities that provide patient privacy protection (such as curtains and privacy processing of bedside card information) can better protect patient privacy.
**RS^l^**		
	RS1	I think it is the duty of doctors to protect patients' privacy.
	RS2	I believe that doctors should protect patients' privacy.
	RS3	I think my sense of responsibility urges me to protect patients' privacy in my daily work.
**PM^m^**		
	PM1	I think doctors' protection of patients' privacy is a requirement of their own professional ethics.
	PM2	I think my sense of professional ethics urges me to protect patients' privacy in my daily work.
	PM3	From education to work, the protection of patients' private information is a professional ethic repeatedly emphasized by doctors.
**EH^n^**		
	EH1	I think doctors should consider the harm of privacy information disclosure from the perspective of patients, to become more aware of protecting the privacy of patients.
	EH2	I have had a personal information disclosure experience as a patient, so I am more aware of protecting the privacy of patients.
	EH3	I think that I can ‘push myself to others’ to protect my patients' privacy in my daily work.
**CF^o^**		
	CF1	I think I have developed a sense of privacy protection in my clinical work.
	CF2	I think I have formed a sense of privacy protection in my teaching.
	CF3	I think I have formed a sense of privacy protection in my own research work.
**BP^p^**		
	BP1	Protect the patient's privacy during surgery or examination, such as curtain pulling and preventing a third party from breaking in.
	BP2	Effectively block the privacy of patients during live operations.
	BP3	Medical observation or teaching requires the consent of the patient.
	BP4	No illegal touch or peek at the patient's privacy.
**IP^q^**		
	IP1	In the situations of outpatient, ward check, case discussion, medical education and observation, the patient shall obtain the consent of the patient himself and take confidentiality measures. The privacy information of the patient shall not be publicized or publicly discussed orally, including the personal information and disease information with identifiable characteristics, such as avoiding calling the full name of the patient loudly, avoiding ‘listening’ or ‘breaking in’ by people other than patients without the consent of the patient.
	IP2	In the face of the condition inquiry, strictly confirm and ask the status of the patient’s condition personnel, confirm as me or with my consent.
	IP3	For patients with special conditions (for example infectious diseases involving privacy), it is necessary to talk to the patients individually.
	IP4	Deliberately disclose and disseminate the privacy of patients without using their duties, such as taking the bedside card test sheets of celebrities to the internet.
	IP5	Protect medical documents such as inspection and medical records without random placing, damage, loss, and prevent theft and being wrongly picked up.
	IP6	Under the unnecessary diagnosis and treatment process, without the consent of the patient, the medical documents shall not be checked, copied, or borrowed during the hospitalization of the patient.
	IP7	Use personal information system account number as required, and login to view patient information without borrowing non-authorised people.
	IP8	Not disclose the privacy information of the patient for any benefit reasons to obtain business, advertise or defraud.
	IP9	When leaving the office seat, protect the pages with patient privacy information and lock the screen of the computer.
	IP10	Scientific research, including the mining of electronic medical record information, whether it is the steps of data acquisition, viewing, processing or analysis, is strictly done to de privacy.
	IP11	In the form of talks or written (case discussion, writing medical treatises, scientific research papers), for example, when communicating and learning on medical social network platform to share typical cases, do well in privacy treatment.
**RP^r^**		
	RP1	Do not disclose information about family members and other personal relationships of any patient.
	RP2	Do not disclose family members and other personal relationship information of any patient on social platforms.
	RP3	Do not verbally promote or publicly discuss family members and other personal relationship information of any patient.

^a^PSE: pperceived severity.

^b^PSU: perceived susceptibility.

^c^IRE: intrinsic rewards.

^d^ERE: extrinsic rewards.

^e^SE: self-efficacy.

^f^RE: response efficacy.

^g^RC: response cost.

^h^SS: supervision support.

^i^IS: information support.

^j^NS: norm support.

^k^ES: environment support.

^l^RS: responsibility.

^m^PM: professional moral.

^n^EH: empathy heart.

^o^CF: consciousness formation.

^p^BP: body privacy.

^q^IP: information privacy.

^r^RP: related privacy.

## Discussion

### Principal Findings

The results of both EFA and CFA revealed the development of awareness and the behavior of public medical institutions in China regarding patients’ privacy protection behavior, which could be measured by the scale proposed in this research. The scale consisted of 18 diverse dimensions, which were theoretically meaningful.

We suggest that the threat assessment process involves 4 dimensions: PSE, PSU, IRE, and ERE. Rogers [21] defines PSE as the judgment of the degree of harmfulness caused by a threat event. PSU refers to the probability of feeling as though a threat event will occur. IRE and ERE both represent the “benefits” introduced by considering hazard factors from the researcher’s own feelings and from the outside world, respectively [22,23]. In this study, PSE included the punishments faced by doctors who disclosed patients’ private information in line with laws and regulations, in addition to the punishment related to hospital norms and the threat of patients’ rights protection. PSU includes the possibility of introducing laws and regulations to punish doctors for disclosing patients’ private information, the possibility of hospitals penalizing doctors for disclosing patients’ private information, and the possibility of patients safeguarding their own rights. IRE includes obtaining financial returns and scientific research achievements, in addition to satisfying vanity by divulging patients’ private information. ERE refers to information doctors hear from the outside world. CA is the assessment of doctors’ ability to protect patients’ privacy, including SE, RE, and RC [22,23]. Throughout this study, SE refers to doctors’ cognition regarding whether they are capable of protecting patients’ privacy. RE refers to the judgment of whether the privacy protection measures undertaken by doctors can protect patient information to an effective degree. RC refers to the cost that doctors are required to pay when taking protective actions, spanning the impact on scientific research and clinical work, that is, doctors’ protection of patients’ privacy will hinder their scientific research work or clinical work.

PMT is used to explain the way in which individuals seek self-protection from a harmful or stressful life [50]. Doctors do not protect patients’ privacy of their own accord. Based on the results of grounded theory, 2 cognitive evaluation processes applicable to this study are summarized: support evaluation and ethical evaluation. Various doctors in the interview revealed that, “…for clinical work, doctors are too busy to notice so much, if possible, hospitals should improve information construction and better protect patient information. In fact, we can make a proposition on how to protect patient privacy in the data flow process...” Throughout this study, support evaluation was predominantly in the context of hospitals. All aspects of hospital support are greatly significant for doctors to successfully carry out patient privacy protection, which can be summarized as SS, IS, NS, and ES. SS means that the hospital must designate a department to perform full-time supervision of patients' privacy protection by doctors, as well as providing training and assessments on privacy protection laws and regulations. IS refers to the improvement in the overall information construction of the hospital and ensuring the privacy and safety of patients are protected. NS includes the establishment and development of a patient privacy protection system, determining the privacy protection process and a reasonable scientific research application system, and carrying out scientific research efficiently on the basis of legal and compliant patient privacy protection. ES refers to improving the medical environment, maintaining the medical order, and providing facilities to prevent patient privacy leakage, for example, curtains, and privacy processing of bedside card information. EA means to analyze and extract relevant factors that promote doctors’ privacy protection behavior according to medical ethics, including RS, PM, and EH. Previous studies have demonstrated that to protect medical privacy, all medical staff who protect others have the responsibility to protect medical privacy. Responsibility ethics is a form of moral thinking that includes other thinking. The lack of moral quality in data application subjects is 1 of the primary subjective factors of anomie of data privacy ethics [51,52]. The original interview stated that “...the main responsibility of doctors is to treat and save patients...” In this study, RS determined that protecting patients’ privacy is both the doctor’s job and social responsibility. The medical ethics of doctors is mentioned in the norms and implementation measures for medical ethics of medical personnel, according to the Ministry of Health [8] issued on December 15, 1988. It discusses the ideological qualities that medical personnel should possess and the sum of the relationship between medical personnel, patients, and society. The implication of medical ethics is to attempt to do everything within your means to be good for patients [53]. “Keeping medical secrets for patients and not divulging patients’ privacy and secrets” is 1 of the provisions mentioned in the code of medical ethics. Similarly, the international code of medical ethics stipulates that “due to the trust of patients, a doctor must absolutely keep patients' privacy” [54]. The original interview stated that “…for my own consideration of professional ethics, I try my best to protect patients’ privacy in clinical and scientific research work...” In this study, the protection of the privacy of patients is a requirement of the doctors’ PM. It is repeatedly emphasized that patients’ private information must be protected by doctors from the educational stage to the work stage. Empathy was first introduced to the field of psychology by the humanistic psychologist Rogers. This refers to an individual understanding of the experience and emotional state of others [55]. Empathetic doctors will convey their understanding to patients [56] and even map the patient’s experience, transforming the patient’s point of view to become a self-centered point of view [57]. According to the analysis of the interview data, a conclusion of this study on empathy was formed, including that doctors can consider the harm of disclosing private information from the perspective of patients and the experience of privacy information disclosure when doctors are patients. Referring to the original text “…imagine that your information will be leaked, commercial and public. Isn't it terrible...do multi-centre research, and I especially emphasize whether the information is desensitized...lack of empathy to protect the patient’s privacy. It's different after thinking about yourself...”

The motivation for protection in PMT has typical characteristics of motivation, which can cause, maintain, and guide activities. Generally, the results of cognitive assessment cause individuals to display greater intention of protective behavior [58]. The concept of protection motivation, in combination with various research scenarios, has evolved into individual intentions of protection behavior, for example, the intention of hot-spring tourists to revisit [59], the intention regarding self-care of elderly patients with chronic diseases [60], and the intention of self-management of diabetic retinopathy [61]. The original interview data discussed that “...the doctors’ work is too busy, and the awareness of patients’ privacy protection is not strong...” Throughout this study, the meaning of doctors’ motivation for patients’ privacy protection in public medical institutions in China is whether doctors develop an awareness of the importance of patient privacy protection.

According to the results of the coding analysis, the privacy protection behavior of doctors includes protecting the private space of patients (BP), protecting the patients’ data (IP), and protecting family members and other personal relations (RP). The protection of BP refers to the protection of the patient’s body privacy by the doctor. Meanwhile, the protection of patient IP refers to the doctor’s protection of the patient’s information privacy. Finally, the protection of patient RP refers to the doctor’s protection of the patient’s associated privacy, including not disclosing or promoting the family members and other personal relationship information about any patient.

Our interviews and scales were created with input from the expected target population, that is, Chinese medical institutions. EFA revealed a unified structure of factor loads without cross loads, allowing a clear interpretation of all potential configurations. CFA confirmed the structure of the scale, meaning the subordinate relationship between each item and the extracted factors was correct. The primary fitting indexes met the requirements of the critical value, and the fitting of CFA was acceptable. It is known that model ﬁt assessment is challenging, considering a small sample size [42], and thus, we agree that further goodness-of-ﬁt assessments with a larger sample size will be necessary in future studies.

### Limitations

This study was subject to various limitations. First, the sample size used was small. Although a general minimum sample size is yet to be deﬁned [34], the authors recommended a sample size at least 5-10 times that of the items [30]. Another limitation was the poor representativeness of samples. In the process of selecting interviewees, the feasibility of obtaining interview samples was considered. Due to the full nature of doctors’ schedules, it was challenging to coordinate a time and place, especially when conducting focus group interviews. Thus, the overall sampling was concentrated in East China. The scale survey was also predominantly based on the principle of convenience sampling, and the regions from which samples were taken mainly included East and North China. Considering the understanding of doctors’ privacy protection behavior by interviewees and respondents, hospitals in different regions have different norms and requirements for doctors’ privacy protection behavior, which would lead to a certain selection bias. In addition, doctors’ privacy protection for patients was not merely a problem of doctors’ behavior but was also due to hospital management. Furthermore, when doctors were interviewed, they might have reservations regarding their true ideas.

### Implications for Future Research

First, we must sample public medical institutions across all 7 regions of China to further verify the reliability and validity of the scale and of doctors’ privacy protection behavior. Subsequently, under the influence of various cognitive mediating factors, doctors’ awareness of patient privacy protection will be affected, resulting in actual privacy protection behavior. The variables will be measured using the scale proposed in this research, and the key factors to promote doctors’ privacy protection behavior will be identified, with the aim of improving it from the perspective of management. In addition to the theoretical model factors, personal factors, including age, gender, educational background, professional title, religious belief, and working years, as well as the environmental policy factors of the region and department of hospitals, also affect doctors’ privacy protection behavior. Nevertheless, to gain additional insights into these relationships and the relative importance of different factors, further research including the scale is needed.

### Conclusion

The theoretical framework and the scale of doctors’ protective behavior of patients’ privacy in public medical institutions fill a crucial gap in the literature and can be used to further the current knowledge of physicians' thought processes and decisions regarding patients’ privacy protection.

## Appendix

Multimedia Appendix 1 The coding results of qualitative research.

Multimedia Appendix 2 The overall cognitive intermediary measurement scale.

Multimedia Appendix 3 Results of EFA. EFA: exploratory factor analysis.

## Abbreviations

AVE: average variance extracted
BP: body privacy
CA: coping appraisal
CF: consciousness formation
CFA: confirmatory factor analysis
CFI: comparative ﬁt index
CPR: composite reliability
CR: critical ratio
EA: ethical appraisal
EFA: exploratory factor analysis
EH: empathy heart
ERE: extrinsic rewards
ES: environment support
IP: information privacy
IRE: intrinsic rewards
IS: information support
KMO: Kaiser-Meyer-Olkin
NS: norm support
PM: professional moral
PMT: protection motivation theory
PSE: perceived severity
PSU: perceived susceptibility
RC: response cost
RE: response efficacy
RMSEA: root-mean-square error of approximation
RP: related privacy
RS: responsibility
SA: support appraisal
SE: self-efficacy
SRMR: standardized root-mean-square residual
SS: supervision support
TA: threat appraisal
TLI: Tucker-Lewis index
