# Supplementary material for: Theoretical Approach and Scale Construction of Patient Privacy Protection Behavior of Doctors in Public Medical Institutions in China: Pilot Development Study
Source: JMIR Form Res. 2022 Dec 14;6(12):e39947. doi: 10.2196/39947 (PMC9798263; doi:10.2196/39947)
Supplement: Multimedia Appendix 1 [file formative_v6i12e39947_app1.docx]

**Appendix 1 The coding results of qualitative research**

It is important to refine the concept of text materials through open, spindle and selective coding. Meanwhile, in order to ensure that the newly constructed theory has sufficient explanatory power concerning the research problems, the theoretical saturation test is essential. We conducted three-level coding on the original interview data and tested the process and results of theoretical saturation. To realise the standardization of the data analysis process, the interview team utilized the qualitative analysis software NVivo 12.0 to assist in the data coding of this study. We conducted both coding analysis and theoretical model construction on ten personal in-depth interviews and one randomly selected focus group interview record. The remaining focus group interview record was reserved for the theoretical saturation test. Through the analysis of open, spindle and selective coding, no new key category of relationship structure was discovered, and no novel constituent factors were established in the six main categories. Thus, the scope of this study could be considered very rich. The internal mechanism of doctors’ privacy protection behavior in the aforementioned China public medical institutions has reached saturation in theory. The coding results of qualitative research are displayed in Tables 1-3.

**Table 1 Open coding categorization**

| **Original Statement Example** | | **Conceptualisation** | **Categorise** |
| --- | --- | --- | --- |
| A1 | ‘... If laws and regulations have some mandatory means, such as the definition of illegality, it may be more binding... the simplest and rough way is to restrict it by laws and regulations. If it is leaked, it will be illegal’. | Enforcement of laws and regulations | B1 threats from laws and regulations |
| A2 | ‘... enhance patients’ awareness of privacy and rights protection. If patients make more noise, doctors will pay more attention to their behavior... if the contradiction between doctors and patients is upgraded due to improper protection of patients’ privacy’. | Doctor patient contradiction | B2 threat of patient rights protection |
| A3 | ‘... hospitals also strengthen management and severely punish such acts. The so-called hero comes out of the stick’. | Hospital standard constraints | B3 threat of hospital norms |
| A4 | ‘... the implementation of relevant legislation is not in place, the punishment and means are missing, and doctors have not taken privacy protection seriously... the illegal cost is relatively low’. | Feel the threat of laws and regulations | B4 feeling the threat of laws and regulations |
| A5 | ‘The key point of doctor-patient contradiction is not privacy disclosure. Doctors are not worried about this, so they don’t pay attention to it’. | Feel the threat of doctor-patient contradiction | B5 feeling the threat of patients’ rights protection |
| A6 | ‘... for the protection of patients’ privacy, the hospital has no compulsory means to implement it... the current hospital rules and regulations should keep up with the hospital system, including the accountability mechanism, are unclear’. | Feel the threat of hospital regulation | B6 feeling the threat of hospital regulation |
| A7 | ‘... it may be the family members of the patient asking if the operation is smooth, the results are good or not, and then handing over a pack of cigarettes’. | Exchange of property for patient information | B7 returns on property |
| A8 | ‘... when building a scientific research database, we will draw on some medical documents during hospitalization to formulate data collection fields’. | The research process and output infringe the privacy information of patients | B8 returns on scientific research results |
| A9 | ‘... I have been in a friend circle to get a star to see a doctor’. | Social network to expose celebrity privacy information to obtain vanity satisfaction | B9 satisfying vanity |
| A10 | ‘... the patient with endocrine Department has a bed card. If the third party gets this information, he can basically know this person. It must be an endocrine related disease. I have heard this information and then sold it to those companies that are making hypoglycaemic drugs. Those companies can pass his name, ask him to push some advertisements on his mobile phone number’.  ‘... these days, it means that there may be economic benefits if there is traffic’. | I heard that others exchanged patient information for property | B10 hearing of property return |
| A11 | ‘... in the scientific report many years ago, the norms of the talks were not strict enough, and there were cases of privacy leakage’.  ‘... talking about the patient’s private information during case discussions such as seminars and symposiums’. | I heard that patients’ privacy information was leaked in the process of scientific research output of others | B11 hearing about the return of scientific research achievements |
| A12 | ‘... maybe some celebrities seek medical treatment. Out of vanity, they will take some photos and send them to the circle of friends to show off’. | Hear others show off their vanity | B12 hearing others show off |
| A13 | ‘...during ward rounds, many patients are in the same room. Sometimes they may ask the patient’s medical history to see the recovery’.  ‘... sometimes the patient will come and ask which bed it is. During our search, the patient may also look at each bed with us. She will see some critical information at the end of the bed on our computer, if we stop him, he will complain about our poor attitude... when there are few people in the hospital, I can better maintain the medical order and let non-patient personnel not enter the consulting room’. | Doctors’ ability to protect patients’ privacy | B13 protection capability assessment |
| A14 | ‘... I've also encountered the scene of patients breaking into the clinic, but my solution is more violent. I scolded the patients directly’.  ‘... It's OK when I'm free. I can't stop it when I'm busy, so I don’t waste time maintaining this treatment order’. | Effectiveness of privacy protection measures taken by doctors for patients | B14 effectiveness of measures |
| A15 | ‘... in previous studies, we also used number codes, but it's really inconvenient. Some patients who need to pay special attention to check can't be found. Our doctors don’t remember the inpatient number and medical record number, but the name can still have an impression. I understand that these divulge the patient’s privacy to a certain extent, but they often happen for convenience’. | Affect scientific research data search | B15 hindering scientific research |
| A16 | ‘... the original data of the hospital may indeed contain sensitive information’.  ‘... in order to avoid information leakage, it is difficult to apply for scientific research data’. | Affect scientific research data acquisition |  |
| A17 | ‘... sometimes when we do data analysis, some data are not very clear. We want to trace some original data. At this time, we can't do it if we don’t check the medical record system, but at the same time, we can see some disease information or sensitive information of patients’. | Analysis of influence on scientific research data |  |
| A18 | ‘... the doctor is too busy to notice so much. If I have to worry about whether there are any other people in the clinic, for example, we need to see 120 to 160 outpatient clinics every day. In fact, I can't ask every patient to enter my clinic, close the door, then ask for a visit, and then let him go out, in this way, I can't finish reading from the morning to two or three in the afternoon’. | Affect clinical work efficiency | B16 hindering clinical work |
| A19 | ‘... for example, multi-disciplinary consultation will discuss patient disease information with other doctors in other departments’.  ‘... some patients have drug allergy history, which is written on the bedside card to remind doctors’.  ‘... when many students go to the physical examination together, they will carry out physical examination for patients, or take a patient as an example for disease analysis during the condition analysis’.  ‘... all the patients in the medical record can query before, and then we divided the rights of these contents of medical history, but there are inconveniences for the division. For example, this person is hospitalized in section A for the first time and the second time to department B, you can only see the relevant medical history of department B, there is no way to see the history of department A, but it is also very troublesome for your diagnosis and treatment. You need to apply to see the relevant medical history of this person in department A’. | Influence the convenience of clinical work |  |
| A20 | ‘... doctors have less work and more work pressure’.  ‘... so much pressure’.  ‘... don’t let doctors have so much pressure anymore’.  ‘... and paying too much attention to these will burden the doctors’ heavy clinical work’. | Increase the workload pressure of doctors |  |
| A21 | ‘... the medical department should pay more attention to these than clinical, so the above are the behaviors of general hospitals, but I believe that the actual compliance will be worse It is still necessary to make clear that the department is responsible for supervision’. | Full-time department supervision and protection of patients’ privacy | B17 supervision by full-time department |
| A22 | ‘... if the hospital has more training in this regard, it should be able to improve’.  ‘... increase the corresponding training’.  ‘... strengthen the training of laws and regulations, so that doctors can learn more about the relevant contents’. | Training on patient privacy protection | B18 training and assessment |
| A23 | ‘... it is necessary to train repeatedly to strengthen memory and regularly evaluate the training content, just as the data security information company assessment’. | Assessment and training contents |  |
| A24 | ‘... hospitals should improve information construction, better protect patient information and how to protect patients’ privacy in the process of data flow’.  ‘... there are many new technologies now, such as artificial intelligence’.  ‘... such as the electronization of bedside card... paperless treatment may be much better’. | Information system privacy and security construction | B19 information privacy security |
| A25 | ‘... the computer can't be directly inserted into the USB flash disk or transmitted to the outside. This is the most basic protection currently visible’.  ‘... taking the operating system as an example, if you don’t work within a few minutes, or haven’t moved for about 10 minutes, it will exit automatically. This may be a protection’. | Information restriction |  |
| A26 | ‘... hospitals can design these systems that involve all aspects of patient privacy details to make doctors have no influence’. | Perfect patient privacy protection system | B20 conventional privacy protection system |
| A27 | ‘... in fact, the hospital management system and implementation are only notified in this area, and there is no good implementation and measures’.  ‘... the hospital can build relevant systems for patient privacy protection, and carry out effective implementation, so as to make clear rewards and punishments'. | Implementation of patient privacy protection system |  |
| A28 | ‘... when doing scientific research, provide a simple application system, fully consider the patient’s privacy and safety, and doctors will not take the initiative to infringe on the patient’s privacy data when the doctor’s data acquisition and compliance are good’. | The scientific research application and data acquisition system are in line with the consideration of patient privacy and safety | B21 scientific research application system |
| A29 | ‘... in the whole scientific research system, the data obtained by doctors are anonymous population data, which can meet the requirements of data analysis and be used to carry out their own scientific research’. | The data processed and analyzed meet the privacy requirements |  |
| A30 | ‘... hospitals also need to improve the environment for medical treatment and improve the order of medical treatment. For example, hundreds of people in large hospitals block up a pile of people at the door of the consulting room every day, so it is difficult to prevent’.  ‘... the order of the hospital needs to be improved’. | Build a good and orderly medical environment | B22 medical environment |
| A31 | ‘... the facilities of the hospital need to be improved’.  ‘... for example, the hardware facilities. You will find that when there are too many patients, you need to see more doctors. He will set up some wooden houses temporarily, and then you can hear every word you say and every word next door clearly’. | Improve privacy protection facilities | B23 perfect facilities |
| A32 | ‘... the doctor’s duty is still to be a doctor’.  ‘... to protect the patient’s privacy is not just a need to be honest’. | Definition of doctors’ job responsibilities | B24 doctor responsibility |
| A33 | ‘... completely rely on the previous social responsibilities and cognition’. | Protecting patients’ privacy is a social responsibility |  |
| A34 | ‘... for my own consideration of professional ethics, I try my best to protect patients’ privacy in clinical and scientific research work’. | Out of the requirements of professional ethics | B25 professional ethics self-discipline |
| A35 | ‘... in terms of privacy protection, we have repeatedly emphasized in our professional ethics’. | Repeated emphasize on professional ethics | B26 professional ethics is emphasized |
| A36 | ‘... imagine that your information will be leaked, commercialized, and made public. Isn't it terrible... do multicentre research, and I especially emphasize whether the information is desensitized’.  ‘... lack of empathy that needs to protect the patient’s privacy. It’s different to think more about yourself’. | Think about the harm of information leakage from the perspective of patients | B27 transposition thinking |
| A37 | ‘... when I gave birth to a child, I received a lot of calls from insurance, mother and baby supplies and photography, which are still very annoying’.  ‘... for example, gossip, who had several children in our hospital, can actually be found’. | As a patient has been leaked privacy information | B28 privacy disclosure experience |
| A38 | ‘... doctors themselves do not have enough concept of maintaining patients’ privacy’.  ‘... they do not have strong awareness of patients’ privacy protection’.  ‘... they need to pay more attention to patients’ privacy protection, so that doctors can form the awareness of maintaining patients’ privacy’. | The formation of doctors’ awareness of patients’ privacy protection | Is B29 aware of patient privacy protection |
| A39 | ‘... the widow of Du (a local celebrity) was admitted to our hospital’.  ‘... many departments came to visit, and everyone came to give diagnosis and treatment advice, which actually affected the patient’s condition’. | Information protection of patients’ family members and other related personnel | B30 patient related personnel information protection |
| A40 | ‘... illegally touching and peeking at the patient’s private parts’.  ‘... the patient’s private parts were not covered by the curtain during nursing’.  ‘... the live broadcast of the operation did not provide privacy treatment for the patient’s body parts’. | Privacy treatment of patients’ body parts | B31 patient’s body and other privacy space protection |
| A41 | ‘... test sheets and bedside cards involve names, hospital numbers, and even more private disease information, such as diagnosis and diet information’. | Patient disease information protection | Data protection of patients and their visits in B32 |
| A42 | ‘... for example, maternity, some people give mobile phone numbers to some advertisements and push anything which are actually sensitive information’. | Patient personal information protection |  |

**Table 2 Main category of spindle coding**

| **Main Category** | **Subcategory** | **Corresponding Category** | **Category Connotation** |  |  |
| --- | --- | --- | --- | --- | --- |
| Threat appraisal | Perceived severity | B1 threats to laws and regulations | The threat of punishment by the law and regulations to doctors’ divulging patients’ privacy. |  |  |
|  |  | B2 threat of patients’ rights protection | Threats caused by patients’ right to protect privacy information disclosure. |  |  |
|  |  | B3 threat of hospital norms | The threat of punishment according to hospital regulations to doctors leaking patients’ privacy. |  |  |
|  | Perceived susceptibility | B4 feeling the threat of laws and regulations | The possibility of punishment by laws and regulations for doctors’ privacy disclosure. |  |  |
|  |  | B5 feeling the threat of patients’ rights protection | The possibility of doctor-patient contradiction caused by the doctors’ privacy disclosure to patients. |  |  |
|  |  | B6 feeling the threat of hospital regulation | The possibility of punishment according to hospital regulations for doctors divulging patients’ privacy. |  |  |
|  | Intrinsic rewards | B7 returns on property | The financial returns of doctors for privacy disclosure of patients. |  |  |
|  |  | B8 returns on scientific research results | The return of scientific research results obtained by doctors for the leakage of patients’ privacy. |  |  |
|  |  | B9 satisfying vanity | The vanity reward of doctors for the privacy disclosure of patients. |  |  |
|  | Extrinsic rewards | B10 return on hearing property | Doctors’ information on the return of property obtained by the privacy disclosure of patients. |  |  |
|  |  | B11 return on hearing and hearing of scientific research achievements | Doctors’ report on the scientific research achievements obtained by the privacy disclosure of patients. |  |  |
|  |  | B12 hearing others show off | Doctors’ experience of vanity rewards from patients’ privacy disclosure. |  |  |
| Coping appraisal | Self-efficacy | B13 protection capability assessment | Doctors’ cognition of whether they are capable of protecting the privacy of patients. |  |  |
|  | Response efficacy | B14 effectiveness of measures | Judge whether the privacy protection measures taken by doctors for patients can effectively protect patient information. |  |  |
|  | Response cost | B15 hindering scientific research | Doctors’ protection of patients’ privacy will hinder their scientific research. |  |  |
|  |  |  |  |  |  |
|  |  |  |  |  |  |
|  |  | B16 hindering clinical work | Doctors’ protection of patients’ privacy will hinder their clinical work. |  |  |
|  |  |  |  |  |  |
|  |  |  |  |  |  |
| Support appraisal | Supervision support | B17 full-time department supervision | It is necessary to designate a department to conduct full-time supervision on the protection of patients’ privacy by doctors. |  |  |
|  |  | B18 training and assessment | According to the legal norms of privacy protection, doctors need to train and assess the contents related to patient privacy protection. |  |  |
|  |  |  |  |  |  |
|  | Information support | B19 information privacy security | Improve the overall information construction of the hospital and protect the privacy and safety of patients. |  |  |
|  |  |  |  |  |  |
|  | Norm support | B20 conventional privacy protection system | Build the system and effective development of patient privacy protection to ensure the privacy protection process. |  |  |
|  |  |  |  |  |  |
|  |  | B21 scientific research application system | Reasonable scientific research application system and carry out scientific research efficiently on the basis of legal and compliant patient privacy protection. |  |  |
|  |  |  |  |  |  |
|  | Environment support | B22 medical environment | Improve the medical environment, maintain the medical order, and reduce the disclosure of patients’ privacy caused by the medical environment. |  |  |
|  |  | B23 perfect facilities | Provide facilities to prevent the disclosure of patients’ privacy, such as curtains, privacy processing of bedside card information. |  |  |
| Ethical appraisal | Responsibility | B24 doctor responsibility | It is both the doctor’s job and social responsibility to protect the patient’s privacy. |  |  |
|  |  |  |  |  |  |
|  | Professional moral | B25 professional ethics self-discipline | Doctors’ protection of patients’ privacy is a requirement of their own professional ethics. |  |  |
|  |  | B26 professional ethics is emphasized | From education to work, the protection of patients’ privacy information is repeatedly emphasized as a doctor. |  |  |
|  | Empathy heart | B27 transposition thinking | Doctors can think about the harm of privacy information disclosure from the perspective of patients. |  |  |
|  |  | B28 privacy disclosure experience | As patients, doctors have had the experience of privacy information being leaked. |  |  |
| Doctors’ motivation to protect patients’ privacy | Consciousness formation | Is B29 aware of patient privacy protection | Do doctors form awareness of patient privacy protection. |  |  |
| Doctors’ behavior of protecting patients’ privacy | Patients’ related privacy protection | B30 patient related personnel information protection | Doctors protect patients’ associated privacy. |  |  |
|  | Patients’ body privacy protection | B31 patient’s body and other privacy space protection | Doctors protect patients’ physical privacy. |  |  |
|  | Patients’ information privacy protection | B32 protection of personal and medical data and information of patients | Doctors protect patients’ information privacy. |  |  |
|  |  |  |  |  |  |
|  |  |  |  |  |  |

**Table 3 Typical relationship structure of PMT theoretical level category**

| **Typical Relationship Structure** | **Connotation of Relationship Structure** |
| --- | --- |
| Threat appraisal (TA) → The motivation of doctors in China public medical institutions to protect patients’ privacy. | TA is a direct factor affecting the motivation of doctors in China public medical institutions to protect patients’ privacy. This includes perceived severity, perceived susceptibility, intrinsic rewards, and extrinsic rewards, all of which directly affect the formation of doctors’ awareness of patients’ privacy protection in China public medical institutions. |
| Coping appraisal (CA)→ The motivation of doctors in China public medical institutions to protect patients’ privacy. | CA is a direct factor affecting the motivation of doctors in China public medical institutions to protect patients’ privacy. This involves the strength of self-efficacy, response efficacy, and response cost, which directly affect the formation of doctors’ awareness of patients’ privacy protection in China public medical institutions. |
| Support appraisal (SA) → The motivation of doctors in China public medical institutions to protect patients’ privacy. | SA is a direct factor affecting the motivation of doctors in China public medical institutions to protect patients’ privacy. It involves the strength of supervision support, information support, norm support, and environment support, all of which directly affect the formation of doctors’ awareness of patients’ privacy protection in China public medical institutions. |
| Ethical appraisal (EA)→ The motivation of doctors in China public medical institutions to protect patients’ privacy. | EA is a direct factor affecting the motivation of doctors in China public medical institutions to protect patients’ privacy. It involves the strength of responsibility, professional moral, and empathy heart, all of which directly affect the formation of doctors' awareness of patients' privacy protection in China public medical institutions. |
| The motivation of doctors in China public medical institutions to protect patients' privacy → behavior of doctors in China public medical institutions to protect patients' privacy. | The motivation of doctors in China public medical institutions to protect patients' privacy is a direct factor affecting doctors' behavior of protecting patients' privacy. This refers to whether doctors in China public medical institutions form awareness of patients' privacy, which directly affects doctors' behavior of protecting patients' privacy in China public medical institutions. |
| 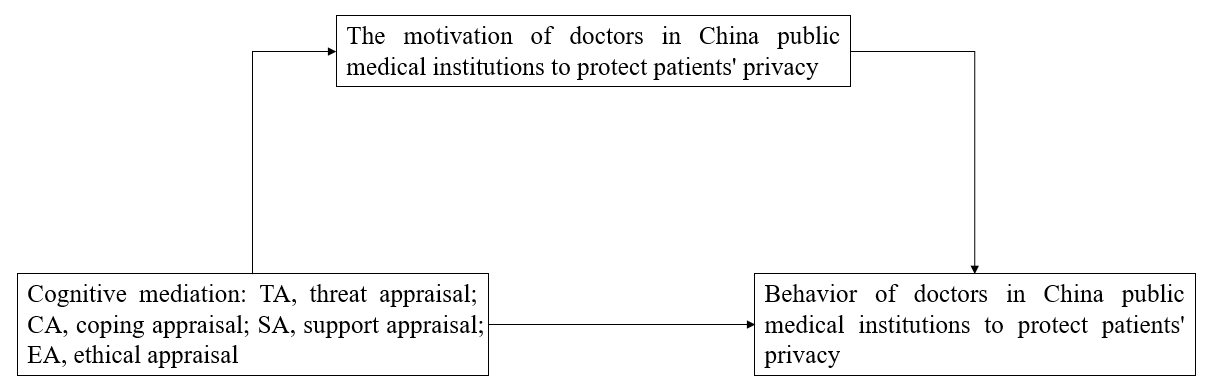 | The motivation of doctors' privacy protection for patients in China public medical institutions plays an intermediary role in the process of cognitive mediation (TA, CA, SA, and EA). These factors all affect doctors' privacy protection behavior for patients (IP, BP, and RP) through the awareness of patients' privacy. |
