# Supplementary material for: Theoretical Approach and Scale Construction of Patient Privacy Protection Behavior of Doctors in Public Medical Institutions in China: Pilot Development Study
Source: JMIR Form Res. 2022 Dec 14;6(12):e39947. doi: 10.2196/39947 (PMC9798263; doi:10.2196/39947)
Supplement: Multimedia Appendix 2 [file formative_v6i12e39947_app2.docx]

**Appendix 2 The overall cognitive intermediary measurement scale**

Cognitive intermediary included four variables: threat, coping, support and ethical appraisals. The threat and coping appraisals in this study were revised and combined with the situation according to previous literature(1-3). As the concepts developed throughout this study, support and ethical appraisals had no available scale, however this would be developed according to the interview data. The overall cognitive intermediary measurement scale is illustrated in Table 4.

**Table 4 Initial scale of cognitive mediators**

| **Variable** | **Variable Dimension** | **Measurement Points** | **Item** | **Reference Source** |
| --- | --- | --- | --- | --- |
| Threat Appraisal (TA) | Perceived Severity (PSE) | Threats to enforcement of laws and regulations | PSE1: I think it is very serious and dangerous that the disclosure of patient privacy information will incur punishment by laws and regulations. | WITTE, et al.(4) |
|  |  | The threat of medical and patient contradictions | PSE2: I think it is very serious and dangerous that the disclosure of patient privacy information will protect patients' rights and deepen the contradiction between doctors and patients. |  |
|  |  | The threat of hospital regulation and restriction | PSE3: I think it is very serious and dangerous that the disclosure of patient privacy information will incur punishment according to the hospital standard system. |  |
|  | Perceived Susceptibility (PSU) | Feel the threat of laws and regulations | PSU1: I think that laws and regulations pay increasing attention to the protection of patients' privacy and have the tendency to make mandatory punishment measures for privacy disclosure. | Mikko Siponen, et al.(5) |
|  |  | Feel the threat of doctor-patient contradiction | PSU2: I think that patients’ awareness of protecting rights is progressively becoming stronger, and the protection of personal privacy is paid increasing attention. The leakage of patient privacy will further deepen the contradiction between doctors and patients. |  |
|  |  | Feel the threat of hospital regulation | PSU3: I think hospitals pay increasing attention to the privacy protection of patients, and the standards and systems will be more and more rigorous, and privacy disclosure incidents will be punishable. |  |
|  | Intrinsic Rewards (IRE) | Exchange of property for patient information | IRE1: I think that the disclosure of patients' privacy can be exchanged for certain financial returns. | Heng Xu, et al.(6) |
|  |  | The research process and output infringe the privacy information of patients | IRE2: I think it is inevitable that patient privacy will be leaked in the process of scientific research output. |  |
|  |  | Social network to expose celebrity privacy information to obtain vanity satisfaction | IRE3: I think meeting celebrities or attending new events at work will ‘get out’ on personal social platforms. |  |
|  | Extrinsic Rewards (ERE) | I heard that others exchanged patient information for property | ERE1: I've heard about the exchange of property through patient privacy information. |  |
|  |  | I heard that patients' privacy information was leaked in the process of scientific research output of others | ERE2: I hear that the easier it is for individuals or institutions to get patient data, the greater the output of scientific research. |  |
|  |  | Hear that others are satisfied with vanity and use patients' privacy to show off | ERE3: I have heard that doctors have exposed some medical information or personal information about celebrities and related people on social platforms. |  |
| Coping Appraisal (CA) | Self efficacy (SE) | Doctors' ability to protect patients' privacy | SE1: I think it's easy for me to protect the privacy of patients. | Johnston, A. C., et al. & Anderson, C. L., et al.(7, 8) |
|  |  |  | SE2: I think it's convenient for me to protect the privacy of patients. |  |
|  |  |  | SE3: I can protect the privacy of patients from being disclosed. |  |
|  | Response Efficacy (RE) | Effectiveness of privacy protection measures taken by doctors for patients | RE1: I think doctors' protection measures to ensure the privacy of patients can effectively prevent the leakage of patients' privacy. | WITTE, et al. & Duy Dang-Pham, et al.(4, 9) |
|  |  |  | RE2: I think the protection measures of doctors can keep patients' privacy in a safe environment. |  |
|  |  |  | RE3: I think the privacy protection measures of doctors for patients can better protect the privacy of patients. |  |
|  | Response Cost (RC) | Affect the search, acquisition, analysis, and output of scientific research data | RC1: I think that paying attention to the protection of patients' privacy will affect the query, acquisition, and analysis of data in my scientific research work and the output of my overall scientific research results. | Vance, A., et al. & Liang, H., et al. & White, G., et al.(10-12) |
|  |  | Affect clinical work efficiency, convenience and increase work pressure | RC2: I think that paying attention to the privacy protection of patients will affect the development and efficiency of work in my clinical or teaching work. |  |
|  |  |  | RC3: I think that paying attention to the privacy protection of patients will increase my work pressure in my clinical or teaching work. |  |
| Support Appraisal (SA) | Supervision Support (SS) | Full time department supervision and protection of patients' privacy | SS1: I think the protection of patients' privacy needs the full-time supervision and management of a hospital department. | Analysis of interview data |
|  |  | Training and assessment of patient privacy protection | SS2: I think it is necessary for the hospital to regularly organize training and assessment according to the laws and regulations related to privacy protection. |  |
|  |  |  | SS3: I think it is necessary for the hospital to regularly organize training and assessment on the hospital system and doctors' protection of patients' privacy. |  |
|  | Information Support (IS) | Information system privacy and security construction | IS1: I think it is necessary to use information technology, artificial intelligence, and other technologies to improve the protection and construction level of the overall informatization of the hospital for the privacy and safety of patients. |  |
|  |  | Information restrictions | IS2: I think it is necessary to carry out reasonable authority management on the information system to protect the patient's private information. |  |
|  |  |  | IS3: I think it is necessary to impose reasonable data transmission restrictions on the information system to protect patients’ private information. |  |
|  | Norm Support (NS) | The construction and implementation of perfect patient privacy protection system | NS1: I think it is necessary to build a patient privacy protection system and carry it out effectively to ensure the rationalization process of patient privacy protection in doctors' work. |  |
|  |  |  | NS2: I think it is necessary to combine the patient privacy protection system with the doctor's daily work, so that the doctor's behavior of protecting the patient's privacy becomes a daily aspect of the work. |  |
|  |  | Reasonable application system of scientific research | NS3: I think it is necessary to formulate a reasonable scientific research application system and conduct scientific research efficiently based on legal and compliant patient privacy protection. |  |
|  | Environment Support (ES) | Build a good and orderly medical environment | ES1: I think improving the medical environment (such as independent consulting room, sound insulation treatment of consulting room) can better protect the privacy of patients. |  |
|  |  |  | ES2: I think it is necessary to maintain the order of medical treatment (for example, prevent irrelevant patients from gathering in the consulting room), which can better protect the privacy of patients. |  |
|  |  | Improve privacy protection facilities | ES3: I think facilities that provide patient privacy protection (such as curtains and privacy processing of bedside card information) can better protect patient privacy. |  |
| Ethical Appraisal (EA) | Responsibility (RS) | Doctors' responsibility | RS1: I think it is the duty of doctors to protect patients' privacy. |  |
|  |  |  | RS2: I think protecting patients' privacy is one of the responsibilities entrusted to doctors by the society. |  |
|  |  |  | RS3: I think my sense of responsibility urges me to protect patients' privacy in my daily work. |  |
|  | Professional Moral (PM) | From professional ethics to their own requirements | PM1: I think doctors' protection of patients' privacy is a requirement of their own professional ethics. |  |
|  |  |  | PM2: I think my sense of professional ethics urges me to protect patients' privacy in my daily work. |  |
|  |  | Repeatedly emphasizing professional ethics | PM3: From education to work, the protection of patients' private information is a professional ethic repeatedly emphasized by doctors. |  |
|  | Empathy Heart (EH) | Thinking about the harm of information being leaked from the perspective of patients | EH1: I think doctors should consider the harm of privacy information disclosure from the perspective of patients, to become more aware of protecting the privacy of patients. |  |
|  |  | The experience of being divulged as a patient of privacy information | EH2: If doctors have the experience of privacy information being leaked when they are patients, they will be more aware of protecting the privacy of patients. |  |
|  |  |  | EH3: I think that I can ‘push myself to others’ to protect my patients' privacy in my daily work. |  |

The motivation of doctors from China’s public medical institutions to protect patients' privacy included consciousness formation. We set the scale of motivation intention in the framework of behavioral theory(13, 14), and subsequently modified and adjusted the dimension of variables obtained from the root theory coding analysis of this study, to ensure that the existing measurement items were more consistent with the research situation. Finally, three items to measure the motivation for protection were generated. The specific measurement items are displayed in Table 5.

**Table 5 Initial scale of doctors' motivation for privacy protection of patients in public health institutions in China**

| **Variable** | **Variable Dimension** | **Measurement Points** | **Item** |
| --- | --- | --- | --- |
| Consciousness Formation (CF) | The formation of doctors' awareness of patients' privacy protection | Whether the awareness of privacy protection of patients is formed | CF1: I think I have developed a sense of privacy protection in my clinical work. |
|  |  |  | CF2: I think I have formed a sense of privacy protection in my teaching. |
|  |  |  | CF3: I think I have formed a sense of privacy protection in my own research work. |

According to the category of patient privacy, we divided the privacy protection of doctors from China’s public medical institutions into three dimensions, the patient's body privacy protection, the patient information privacy protection, and the patient related privacy protection behavior. Based on the literature summary(15-17) and the coding analysis results of the root theory in the previous article, 20 questions were proposed. The specific measurement items are listed in Table 6.

**Table 6 Initial scale of doctors' privacy protection behavior of China’s public medical institutions**

| **Variable** | **Item** |
| --- | --- |
| Body Privacy (BP) | BP1: Protect the patient's privacy during surgery or examination, such as curtain pulling and preventing a third party from breaking in. |
|  | BP2: Effectively block the privacy of patients during live operations. |
|  | BP3: No illegal touch or peek at the patient's privacy. |
| Information Privacy (IP) | IP1: In the situations of outpatient, ward check, case discussion, medical education and observation, the patient shall obtain the consent of the patient himself and take confidentiality measures. The privacy information of the patient shall not be publicized or publicly discussed orally, including the personal information and disease information with identifiable characteristics, such as avoiding calling the full name of the patient loudly, avoiding ‘listening’ or ‘breaking in’ by people other than patients without the consent of the patient. |
|  | IP2: In the face of the condition inquiry, strictly confirm and ask the status of the patient’s condition personnel, confirm as me or with my consent. |
|  | IP3: For patients with special conditions (for example infectious diseases involving privacy), it is necessary to talk to the patients individually. |
|  | IP4: Deliberately disclose and disseminate the privacy of patients without using their duties, such as taking the bedside card test sheets of celebrities to the internet. |
|  | IP5: Protect medical documents such as inspection and medical records without random placing, damage, loss, and prevent theft and being wrongly picked up. |
|  | IP6: Under the unnecessary diagnosis and treatment process, without the consent of the patient, the medical documents shall not be checked, copied, or borrowed during the hospitalization of the patient. |
|  | IP7: Use personal information system account number as required, and login to view patient information without borrowing non-authorised people. |
|  | IP8: Not disclose the privacy information of the patient for any benefit reasons to obtain business, advertise or defraud. |
|  | IP9: When leaving the office seat, protect the pages with patient privacy information and lock the screen of the computer. |
|  | IP10: Scientific research, including the mining of electronic medical record information, whether it is the steps of data acquisition, viewing, processing or analysis, is strictly done to de privacy. |
|  | IP11: In the form of talks or written (case discussion, writing medical treatises, scientific research papers), for example, when communicating and learning on medical social network platform to share typical cases, do well in privacy treatment. |
| Related Privacy (RP) | RP1: Do not disclose information about family members and other personal relationships of any patient. |
|  | RP2: Do not disclose family members and other personal relationship information of any patient on social platforms. |
|  | RP3: Do not verbally promote or publicly discuss family members and other personal relationship information of any patient. |

**Reference**

1. Huafang C, Fei X. Study on Influencing Factors of nurses' privacy protection behavior in electronic medical record. Journal of Medical Informatics. 2021;42(4):6

2. dingjuan W. Privacy concerns and influencing factors of medical data in the context of big data: An Empirical Study Based on protection motivation theory. Journal of Henan Normal University: Philosophy and Social Sciences Edition. 2020;47(5):7

3. Zhu Guang LF, Bian Shuying. Research on the use intention of smart medicine driven by privacy concern. Library Forum. 2021:1-16

4. WITTE, KIM. Predicting Risk Behaviors: Development and Validation of a Diagnostic Scale. Journal of Health Communication. 1996;1(4):317-42

5. Siponen M, Mahmood MA, Pahnila S. Employees' adherence to information security policies: An exploratory field study. Information & Management. 2014;51(2):217-24

6. Heng Xu XRL, John M. Ca Rroll, Mary Beth Rosson. The personalization privacy paradox: An exploratory study of decision making process for location-aware marketing. Decision Support Systems. 2011;51(1):42-52

7. Anderson CL, Agarwal R. Practicing Safe Computing: A Multimedia Empirical Examination of Home Computer User Security Behavioral Intentions. Mis Quarterly. 2010;34(3):613-43

8. Johnston AC, Warkentin M. Fear Appeals and Information Security Behaviors: An Empirical Study. MIS Quarterly. 2010;34(3):549-66

9. Dang-Pham D, Pittayachawan S. Comparing intention to avoid malware across contexts in a BYOD-enabled Australian university: A Protection Motivation Theory approach. Computers & Security. 2015;48(feb.):281-97

10. White GL, Ju L. Thinking Globally: Incorporating an International Component in Information Security Curriculums. 2014

11. Liang H, Xue YL. Understanding Security Behaviors in Personal Computer Usage: A Threat Avoidance Perspective. Journal of the Association for Information Systems. 2010;11(7):394-413

12. Vance A, Siponen M, Pahnila S. Motivating IS security compliance: Insights from Habit and Protection Motivation Theory. Information & Management. 2012;49(3-4):190-8

13. Ma CC, Kuo KM, Alexander JW. A survey-based study of factors that motivate nurses to protect the privacy of electronic medical records. Bmc Medical Informatics & Decision Making. 2016;16(1):13

14. Bansal G, Zahedi FM, Gefen D. The impact of personal dispositions on information sensitivity, privacy concern and trust in disclosing health information online. Decision Support Systems. 2010;49(2):138-50

15. Li Runhua LY. Medical Ethics (Vol. 33)2003.

16. health Mo. Norms and implementation measures of medical ethics of medical personnel. Hospital management in China. 1989;9(3)

17. China PsGotpsRo. Law of the people's Republic of China on licensed doctors (2005) [Available from: <http://www.gov.cn/banshi/2005-08/01/content_18970.htm>.
