# Supplementary material for: Theoretical Approach and Scale Construction of Patient Privacy Protection Behavior of Doctors in Public Medical Institutions in China: Pilot Development Study
Source: JMIR Form Res. 2022 Dec 14;6(12):e39947. doi: 10.2196/39947 (PMC9798263; doi:10.2196/39947)
Supplement: Multimedia Appendix 3 [file formative_v6i12e39947_app3.docx]

**Multimedia Appendix 3.** Results of EFA^a^.

| Variable and item | | Component | | | | | | | | | | | | | | | | | |
| --- | --- | --- | --- | --- | --- | --- | --- | --- | --- | --- | --- | --- | --- | --- | --- | --- | --- | --- | --- |
|  |  | 1 | 2 | 3 | 4 | 5 | 6 | 7 | 8 | 9 | 10 | 11 | 12 | 13 | 14 | 15 | 16 | 17 | 18 |
|  |  |  |  |  |  |  |  |  |  |  |  |  |  |  |  |  |  |  |  |
| **PSE^b^ (cumulative explanatory variance=3.639, Cronbach α=.817)** | | | | | | | | | | | | | | | | | | | |
|  | PSE1 | N/A^c^ | N/A | N/A | N/A | N/A | 0.795 | N/A | N/A | N/A | N/A | N/A | N/A | N/A | N/A | N/A | N/A | N/A | N/A |
|  | PSE2 | N/A | N/A | N/A | N/A | N/A | 0.826 | N/A | N/A | N/A | N/A | N/A | N/A | N/A | N/A | N/A | N/A | N/A | N/A |
|  | PSE3 | N/A | N/A | N/A | N/A | N/A | 0.847 | N/A | N/A | N/A | N/A | N/A | N/A | N/A | N/A | N/A | N/A | N/A | N/A |
| **PSU^d^ (cumulative explanatory variance=7.236, Cronbach α=.84)** | | | | | | | | | | | | | | | | | | | |
|  | PSU1 | N/A | N/A | N/A | N/A | N/A | N/A | N/A | 0.809 | N/A | N/A | N/A | N/A | N/A | N/A | N/A | N/A | N/A | N/A |
|  | PSU2 | N/A | N/A | N/A | N/A | N/A | N/A | N/A | 0.801 | N/A | N/A | N/A | N/A | N/A | N/A | N/A | N/A | N/A | N/A |
|  | PSU3 | N/A | N/A | N/A | N/A | N/A | N/A | N/A | 0.816 | N/A | N/A | N/A | N/A | N/A | N/A | N/A | N/A | N/A | N/A |
| **IRE^e^ (cumulative explanatory variance=10.717, Cronbach α=.789)** | | | | | | | | | | | | | | | | | | | |
|  | IRE1 | N/A | N/A | N/A | N/A | N/A | N/A | N/A | N/A | N/A | N/A | N/A | N/A | N/A | N/A | N/A | 0.841 | N/A | N/A |
|  | IRE2 | N/A | N/A | N/A | N/A | N/A | N/A | N/A | N/A | N/A | N/A | N/A | N/A | N/A | N/A | N/A | 0.724 | N/A | N/A |
|  | IRE3 | N/A | N/A | N/A | N/A | N/A | N/A | N/A | N/A | N/A | N/A | N/A | N/A | N/A | N/A | N/A | 0.837 | N/A | N/A |
| **ERE^f^ (cumulative explanatory variance=14.38, Cronbach α=.777)** | | | | | | | | | | | | | | | | | | | |
|  | ERE1 | N/A | N/A | N/A | 0.785 | N/A | N/A | N/A | N/A | N/A | N/A | N/A | N/A | N/A | N/A | N/A | N/A | N/A | N/A |
|  | ERE2 | N/A | N/A | N/A | 0.797 | N/A | N/A | N/A | N/A | N/A | N/A | N/A | N/A | N/A | N/A | N/A | N/A | N/A | N/A |
|  | ERE3 | N/A | N/A | N/A | 0.759 | N/A | N/A | N/A | N/A | N/A | N/A | N/A | N/A | N/A | N/A | N/A | N/A | N/A | N/A |
| **SE^g^ (cumulative explanatory variance=18.063, Cronbach α=.818)** | | | | | | | | | | | | | | | | | | | |
|  | SE1 | N/A | N/A | 0.833 | N/A | N/A | N/A | N/A | N/A | N/A | N/A | N/A | N/A | N/A | N/A | N/A | N/A | N/A | N/A |
|  | SE2 | N/A | N/A | 0.824 | N/A | N/A | N/A | N/A | N/A | N/A | N/A | N/A | N/A | N/A | N/A | N/A | N/A | N/A | N/A |
|  | SE3 | N/A | N/A | 0.812 | N/A | N/A | N/A | N/A | N/A | N/A | N/A | N/A | N/A | N/A | N/A | N/A | N/A | N/A | N/A |
| **RE^h^ (cumulative explanatory variance=21.597, Cronbach α=.794)** | | | | | | | | | | | | | | | | | | | |
|  | RE1 | N/A | N/A | N/A | N/A | N/A | N/A | N/A | N/A | N/A | N/A | 0.833 | N/A | N/A | N/A | N/A | N/A | N/A | N/A |
|  | RE2 | N/A | N/A | N/A | N/A | N/A | N/A | N/A | N/A | N/A | N/A | 0.835 | N/A | N/A | N/A | N/A | N/A | N/A | N/A |
|  | RE3 | N/A | N/A | N/A | N/A | N/A | N/A | N/A | N/A | N/A | N/A | 0.779 | N/A | N/A | N/A | N/A | N/A | N/A | N/A |
| **RC^i^ (cumulative explanatory variance=25.137, Cronbach α=.792)** | | | | | | | | | | | | | | | | | | | |
|  | RC1 | N/A | N/A | N/A | N/A | N/A | N/A | N/A | N/A | N/A | 0.726 | N/A | N/A | N/A | N/A | N/A | N/A | N/A | N/A |
|  | RC2 | N/A | N/A | N/A | N/A | N/A | N/A | N/A | N/A | N/A | 0.856 | N/A | N/A | N/A | N/A | N/A | N/A | N/A | N/A |
|  | RC3 | N/A | N/A | N/A | N/A | N/A | N/A | N/A | N/A | N/A | 0.835 | N/A | N/A | N/A | N/A | N/A | N/A | N/A | N/A |
| **SS^j^ (cumulative explanatory variance=28.668, Cronbach α=.773)** | | | | | | | | | | | | | | | | | | | |
|  | SS1 | N/A | N/A | N/A | N/A | N/A | N/A | N/A | N/A | N/A | N/A | N/A | 0.769 | N/A | N/A | N/A | N/A | N/A | N/A |
|  | SS2 | N/A | N/A | N/A | N/A | N/A | N/A | N/A | N/A | N/A | N/A | N/A | 0.779 | N/A | N/A | N/A | N/A | N/A | N/A |
|  | SS3 | N/A | N/A | N/A | N/A | N/A | N/A | N/A | N/A | N/A | N/A | N/A | 0.791 | N/A | N/A | N/A | N/A | N/A | N/A |
| **IS^k^ (cumulative explanatory variance=32.221, Cronbach α=.797)** | | | | | | | | | | | | | | | | | | | |
|  | IS1 | N/A | N/A | N/A | N/A | N/A | N/A | N/A | N/A | 0.824 | N/A | N/A | N/A | N/A | N/A | N/A | N/A | N/A | N/A |
|  | IS2 | N/A | N/A | N/A | N/A | N/A | N/A | N/A | N/A | 0.769 | N/A | N/A | N/A | N/A | N/A | N/A | N/A | N/A | N/A |
|  | IS3 | N/A | N/A | N/A | N/A | N/A | N/A | N/A | N/A | 0.803 | N/A | N/A | N/A | N/A | N/A | N/A | N/A | N/A | N/A |
| **NS^l^ (cumulative explanatory variance=35.855, Cronbach α=.809)** | | | | | | | | | | | | | | | | | | | |
|  | NS1 | N/A | N/A | N/A | N/A | N/A | N/A | 0.769 | N/A | N/A | N/A | N/A | N/A | N/A | N/A | N/A | N/A | N/A | N/A |
|  | NS2 | N/A | N/A | N/A | N/A | N/A | N/A | 0.824 | N/A | N/A | N/A | N/A | N/A | N/A | N/A | N/A | N/A | N/A | N/A |
|  | NS3 | N/A | N/A | N/A | N/A | N/A | N/A | 0.838 | N/A | N/A | N/A | N/A | N/A | N/A | N/A | N/A | N/A | N/A | N/A |
| **ES^m^ (cumulative explanatory variance=39.374, Cronbach α=.796)** | | | | | | | | | | | | | | | | | | | |
|  | ES1 | N/A | N/A | N/A | N/A | N/A | N/A | N/A | N/A | N/A | N/A | N/A | N/A | N/A | 0.791 | N/A | N/A | N/A | N/A |
|  | ES2 | N/A | N/A | N/A | N/A | N/A | N/A | N/A | N/A | N/A | N/A | N/A | N/A | N/A | 0.792 | N/A | N/A | N/A | N/A |
|  | ES3 | N/A | N/A | N/A | N/A | N/A | N/A | N/A | N/A | N/A | N/A | N/A | N/A | N/A | 0.807 | N/A | N/A | N/A | N/A |
| **RS^n^ (cumulative explanatory variance=42.886, Cronbach α=.782)** | | | | | | | | | | | | | | | | | | | |
|  | RS1 | N/A | N/A | N/A | N/A | N/A | N/A | N/A | N/A | N/A | N/A | N/A | N/A | N/A | N/A | 0.786 | N/A | N/A | N/A |
|  | RS2 | N/A | N/A | N/A | N/A | N/A | N/A | N/A | N/A | N/A | N/A | N/A | N/A | N/A | N/A | 0.806 | N/A | N/A | N/A |
|  | RS3 | N/A | N/A | N/A | N/A | N/A | N/A | N/A | N/A | N/A | N/A | N/A | N/A | N/A | N/A | 0.817 | N/A | N/A | N/A |
| **PM^o^ (cumulative explanatory variance=46.35, Cronbach α=.781)** | | | | | | | | | | | | | | | | | | | |
|  | PM1 | N/A | N/A | N/A | N/A | N/A | N/A | N/A | N/A | N/A | N/A | N/A | N/A | N/A | N/A | N/A | N/A | 0.822 | N/A |
|  | PM2 | N/A | N/A | N/A | N/A | N/A | N/A | N/A | N/A | N/A | N/A | N/A | N/A | N/A | N/A | N/A | N/A | 0.837 | N/A |
|  | PM3 | N/A | N/A | N/A | N/A | N/A | N/A | N/A | N/A | N/A | N/A | N/A | N/A | N/A | N/A | N/A | N/A | 0.77 | N/A |
| **EH^p^ (cumulative explanatory variance=49.879, Cronbach α=.769)** | | | | | | | | | | | | | | | | | | | |
|  | EH1 | N/A | N/A | N/A | N/A | N/A | N/A | N/A | N/A | N/A | N/A | N/A | N/A | 0.819 | N/A | N/A | N/A | N/A | N/A |
|  | EH2 | N/A | N/A | N/A | N/A | N/A | N/A | N/A | N/A | N/A | N/A | N/A | N/A | 0.807 | N/A | N/A | N/A | N/A | N/A |
|  | EH3 | N/A | N/A | N/A | N/A | N/A | N/A | N/A | N/A | N/A | N/A | N/A | N/A | 0.781 | N/A | N/A | N/A | N/A | N/A |
| **CF^q^ (cumulative explanatory variance=53.277, Cronbach α=.754)** | | | | | | | | | | | | | | | | | | | |
|  | CF1 | N/A | N/A | N/A | N/A | N/A | N/A | N/A | N/A | N/A | N/A | N/A | N/A | N/A | N/A | N/A | N/A | N/A | 0.801 |
|  | CF2 | N/A | N/A | N/A | N/A | N/A | N/A | N/A | N/A | N/A | N/A | N/A | N/A | N/A | N/A | N/A | N/A | N/A | 0.782 |
|  | CF3 | N/A | N/A | N/A | N/A | N/A | N/A | N/A | N/A | N/A | N/A | N/A | N/A | N/A | N/A | N/A | N/A | N/A | 0.841 |
| **BP^r^ (cumulative explanatory variance=57.676, Cronbach α=.831)** | | | | | | | | | | | | | | | | | | | |
|  | BP1 | N/A | 0.82 | N/A | N/A | N/A | N/A | N/A | N/A | N/A | N/A | N/A | N/A | N/A | N/A | N/A | N/A | N/A | N/A |
|  | BP2 | N/A | 0.815 | N/A | N/A | N/A | N/A | N/A | N/A | N/A | N/A | N/A | N/A | N/A | N/A | N/A | N/A | N/A | N/A |
|  | BP3 | N/A | 0.782 | N/A | N/A | N/A | N/A | N/A | N/A | N/A | N/A | N/A | N/A | N/A | N/A | N/A | N/A | N/A | N/A |
|  | BP4 | N/A | 0.814 | N/A | N/A | N/A | N/A | N/A | N/A | N/A | N/A | N/A | N/A | N/A | N/A | N/A | N/A | N/A | N/A |
| **IP^s^ (cumulative explanatory variance=67.845, Cronbach α=.926)** | | | | | | | | | | | | | | | | | | | |
|  | IP1 | 0.751 | N/A | N/A | N/A | N/A | N/A | N/A | N/A | N/A | N/A | N/A | N/A | N/A | N/A | N/A | N/A | N/A | N/A |
|  | IP2 | 0.75 | N/A | N/A | N/A | N/A | N/A | N/A | N/A | N/A | N/A | N/A | N/A | N/A | N/A | N/A | N/A | N/A | N/A |
|  | IP3 | 0.765 | N/A | N/A | N/A | N/A | N/A | N/A | N/A | N/A | N/A | N/A | N/A | N/A | N/A | N/A | N/A | N/A | N/A |
|  | IP4 | 0.755 | N/A | N/A | N/A | N/A | N/A | N/A | N/A | N/A | N/A | N/A | N/A | N/A | N/A | N/A | N/A | N/A | N/A |
|  | IP5 | 0.766 | N/A | N/A | N/A | N/A | N/A | N/A | N/A | N/A | N/A | N/A | N/A | N/A | N/A | N/A | N/A | N/A | N/A |
|  | IP6 | 0.82 | N/A | N/A | N/A | N/A | N/A | N/A | N/A | N/A | N/A | N/A | N/A | N/A | N/A | N/A | N/A | N/A | N/A |
|  | IP7 | 0.766 | N/A | N/A | N/A | N/A | N/A | N/A | N/A | N/A | N/A | N/A | N/A | N/A | N/A | N/A | N/A | N/A | N/A |
|  | IP8 | 0.709 | N/A | N/A | N/A | N/A | N/A | N/A | N/A | N/A | N/A | N/A | N/A | N/A | N/A | N/A | N/A | N/A | N/A |
|  | IP9 | 0.788 | N/A | N/A | N/A | N/A | N/A | N/A | N/A | N/A | N/A | N/A | N/A | N/A | N/A | N/A | N/A | N/A | N/A |
|  | IP10 | 0.733 | N/A | N/A | N/A | N/A | N/A | N/A | N/A | N/A | N/A | N/A | N/A | N/A | N/A | N/A | N/A | N/A | N/A |
|  | IP11 | 0.719 | N/A | N/A | N/A | N/A | N/A | N/A | N/A | N/A | N/A | N/A | N/A | N/A | N/A | N/A | N/A | N/A | N/A |
| **RP^t^ (cumulative explanatory variance=71.493, Cronbach α=.801)** | | | | | | | | | | | | | | | | | | | |
|  | RP1 | N/A | N/A | N/A | N/A | 0.831 | N/A | N/A | N/A | N/A | N/A | N/A | N/A | N/A | N/A | N/A | N/A | N/A | N/A |
|  | RP2 | N/A | N/A | N/A | N/A | 0.845 | N/A | N/A | N/A | N/A | N/A | N/A | N/A | N/A | N/A | N/A | N/A | N/A | N/A |
|  | RP3 | N/A | N/A | N/A | N/A | 0.816 | N/A | N/A | N/A | N/A | N/A | N/A | N/A | N/A | N/A | N/A | N/A | N/A | N/A |

^a^EFA: exploratory factor analysis.

^b^PSE: perceived severity.

^c^N/A: not applicable.

^d^PSU: perceived susceptibility.

^e^IRE: intrinsic rewards.

^f^ERE: extrinsic rewards.

^g^SE: self-efficacy.

^h^RE: response efficacy.

^i^RC: response cost.

^j^SS: supervision support.

^k^IS: information support.

^l^NS: norm support.

^m^ES: environment support.

^n^RS: responsibility.

^o^PM: professional moral.

^p^EH: empathy heart.

^q^CF: consciousness formation.

^r^BP: body privacy.

^s^IP: information privacy.

^t^RP: related privacy.
